# In vivo labeling reveals continuous trafficking of TCF-1^+^ T cells between tumor and lymphoid tissue

**DOI:** 10.1084/jem.20210749

**Published:** 2022-04-26

**Authors:** Zhi Li, Zewen K. Tuong, Isaac Dean, Claire Willis, Fabrina Gaspal, Rémi Fiancette, Suaad Idris, Bethany Kennedy, John R. Ferdinand, Ana Peñalver, Mia Cabantous, Syed Murtuza Baker, Jeremy W. Fry, Gianluca Carlesso, Scott A. Hammond, Simon J. Dovedi, Matthew R. Hepworth, Menna R. Clatworthy, David R. Withers

**Affiliations:** 1 Institute of Immunology and Immunotherapy, College of Medical and Dental Sciences, University of Birmingham, Birmingham, UK; 2 Molecular Immunity Unit, Department of Medicine, University of Cambridge, Cambridge, UK; 3 Cellular Genetics, Wellcome Trust Sanger Institute, Hinxton, UK; 4 Division of Informatics, Imaging & Data Science, Faculty of Biology, Medicine and Health, the University of Manchester, Manchester Academic Health Science Centre, Manchester, UK; 5 ProImmune Ltd., The Magdalen Centre, Oxford Science Park, Oxford, UK; 6 Early Oncology R&D, AstraZeneca, Gaithersburg, MD; 7 Early Oncology R&D, AstraZeneca, Cambridge, UK; 8 Lydia Becker Institute of Immunology and Inflammation, Division of Infection, Immunity and Respiratory Medicine, Faculty of Biology, Medicine and Health, the University of Manchester, Manchester Academic Health Science Centre, Manchester, UK

## Abstract

Improving the efficacy of immune checkpoint therapies will require a better understanding of how immune cells are recruited and sustained in tumors. Here, we used the photoconversion of the tumor immune cell compartment to identify newly entering lymphocytes, determine how they change over time, and investigate their egress from the tumor. Combining single-cell transcriptomics and flow cytometry, we found that while a diverse mix of CD8 T cell subsets enter the tumor, all CD8 T cells retained within this environment for more than 72 h developed an exhausted phenotype, revealing the rapid establishment of this program. Rather than forming tumor-resident populations, non-effector subsets, which express TCF-1 and include memory and stem-like cells, were continuously recruited into the tumor, but this recruitment was balanced by concurrent egress to the tumor-draining lymph node. Thus, the TCF-1^+^ CD8 T cell niche in tumors is highly dynamic, with the circulation of cells between the tumor and peripheral lymphoid tissue to bridge systemic and intratumoral responses.

## Teaser

How intratumoral T cell subsets differ in their recruitment or retention is unclear. In this study, photoconversion is used to temporally label tumor-infiltrating lymphocytes, revealing the continuous migration of TCF-1^+^ T cells between the tumor and draining lymphoid tissue.

## Graphical Abstract

**Figure:**
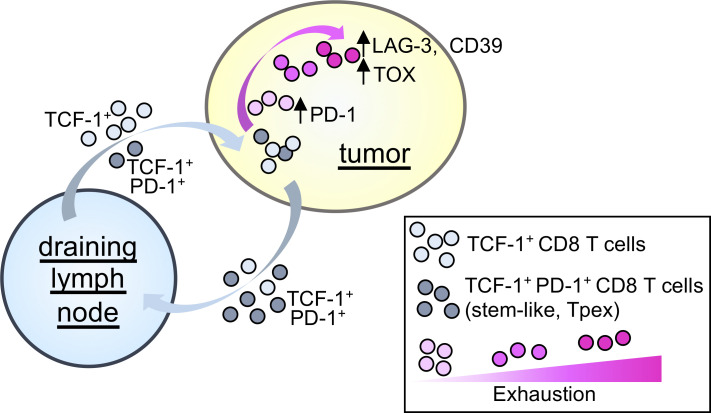

## Introduction

T cell infiltration is predictive of patient survival and the response to immunotherapy for many cancers (Azimi et al., 2012; Galon et al., 2006; Herbst et al., 2014; Naito et al., 1998; Tumeh et al., 2014). A defining feature of intratumoral T cells is an altered or exhausted phenotype, first characterized in chronic viral infections (Wherry et al., 2007), where persistent exposure to antigen (Ag) results in defective T cell responses (Gallimore et al., 1998; Zajac et al., 1998). Compared with conventional effector cells, exhausted CD8 T cells have impaired functions, highly express multiple inhibitory receptors including PD-1, LAG-3, and TIM-3, and have distinct transcriptional and metabolic profiles (McLane et al., 2019). Crucially, antibodies (Abs) against PD-1 reinvigorated the CD8 T cell response during chronic lymphocytic choriomeningitis virus (LCMV) infection demonstrating that exhaustion could be overcome (Barber et al., 2006; Pauken et al., 2016), and preclinical cancer models established the efficacy of targeting the PD-1:PD-L1 pathway to limit tumor growth (Blank et al., 2004; Iwai et al., 2005). Targeting the PD-1:PD-L1 pathway or CTLA-4 to enhance anti-tumor T cell activity, termed immune checkpoint blockade (ICB) therapy, results in striking clinical responses in some tumors including melanoma, renal cell carcinoma, and non-small cell lung cancer (Brahmer et al., 2012; Hodi et al., 2010; Topalian et al., 2012). However, only a minority of patients robustly and durably respond to these therapies, and while combined targeting of PD-1 and CTLA-4 enhances tumor regression, it is also associated with significantly more adverse events (Chae et al., 2018; Curran et al., 2010; Postow et al., 2015).

While T cell infiltration predicts responsiveness to ICB (Daud et al., 2016; Jiang et al., 2018; Teng et al., 2015), the origin and fate of tumor-infiltrating lymphocytes (TILs) and how specific therapies impact this remain incompletely understood. Analysis of chromatin accessibility revealed two dysfunctional CD8 T cell states, an early “plastic” state and a second more “locked” state that then persisted and was resistant to reprogramming (Philip et al., 2017). The effector T cell response appears to be sustained by a “stem-like” population of TCF-1^+^ PD-1^+^ CD8 T cells that are required for tumor control in response to immunotherapy (Brummelman et al., 2018; He et al., 2016; Im et al., 2016; Jansen et al., 2019; Kurtulus et al., 2019; Sade-Feldman et al., 2018; Siddiqui et al., 2019; Utzschneider et al., 2016). While considered resident within lymphoid tissues following LCMV infection (Im et al., 2020), the presence of TCF-1^+^ PD-1^+^ cells within tumors indicates different migratory potential during anti-tumor responses. Notably, these cells were not evenly distributed throughout human tumors; rather, they were principally located within a specific niche (Jansen et al., 2019). Whether TCF-1^+^ PD-1^+^ CD8 T cells become resident within tumors is unclear; however, current data indicate that intratumoral expansion of TCF-1^+^ PD-1^+^ CD8 T cells is critical for tumor regression. Elegant in vivo imaging revealed that a feedback loop of enhanced intratumoral T cell IFNγ expression stimulated local IL-12 production by dendritic cells (DCs) to then fully drive the CD8 anti-tumor T cell response (Garris et al., 2018). However, alongside effects within the tumor microenvironment, a critical role for systemic immune responses in tumor eradication has also been proposed (Spitzer et al., 2017; Wu et al., 2020). Notably, the use of low-dose anti–PD-L1 Abs to specifically target interactions within the draining LN (dLN) still caused tumor regression that was further dependent upon T cell egress and trafficking to the tumor (Dammeijer et al., 2020). These data, in particular, raise the questions of where effector T cell activation and expansion occur in response to anti–PD-L1 treatment and the role of intratumoral TCF-1^+^ PD-1^+^ CD8 T cells in these responses.

To date, studies of the anti-tumor response have lacked approaches that enable dynamic analyses of TILs to track cellular changes over time, in conjunction with delineating the source and destination of specific populations. Immuno-PET has been used to longitudinally assess immune infiltrates and correlate CD8 T cell distribution with successful control of tumor growth (Rashidian et al., 2017; Rashidian et al., 2019), but this approach lacks detailed descriptions of changes in phenotype and function. To directly investigate the in vivo trafficking of T cells into and out of tumors, here we have exploited photoactivatable Kaede transgenic mice to enable specific labeling of tumor immune cells and direct analysis of their migration (Houston et al., 2016; Marriott et al., 2017; Morton et al., 2014; Tomura et al., 2014; Tomura et al., 2008; Ugur et al., 2014). Using single-cell RNA sequencing (scRNA-seq), we defined the temporal transcriptional dynamics of intratumoral T cells and observed that all CD8 T cells retained in the tumor rapidly developed an exhausted phenotype within a matter of days. While a heterogeneous mix of TCF-1^+^ CD8 T cells were evident amongst newly recruited TILs, these populations, including the TCF-1^+^ PD-1^+^ population were not retained over time in the tumor and were abundant amongst the T cells that egressed to the dLN. We further showed that the blockade of PD-L1 resulted in the enhanced activation of effectors cells newly arrived in the tumor, alongside reinvigoration of exhausted CD8 T cells retained within the tumor. Collectively, our data provide the first detailed dynamic analysis of changes in TILs, revealing subset-specific trafficking properties and the rapid induction of T cell exhaustion amongst tumor-retained populations.

## Results

### Labeling of the tumor immune compartment to track how TILs change over time

**Figure 1.**
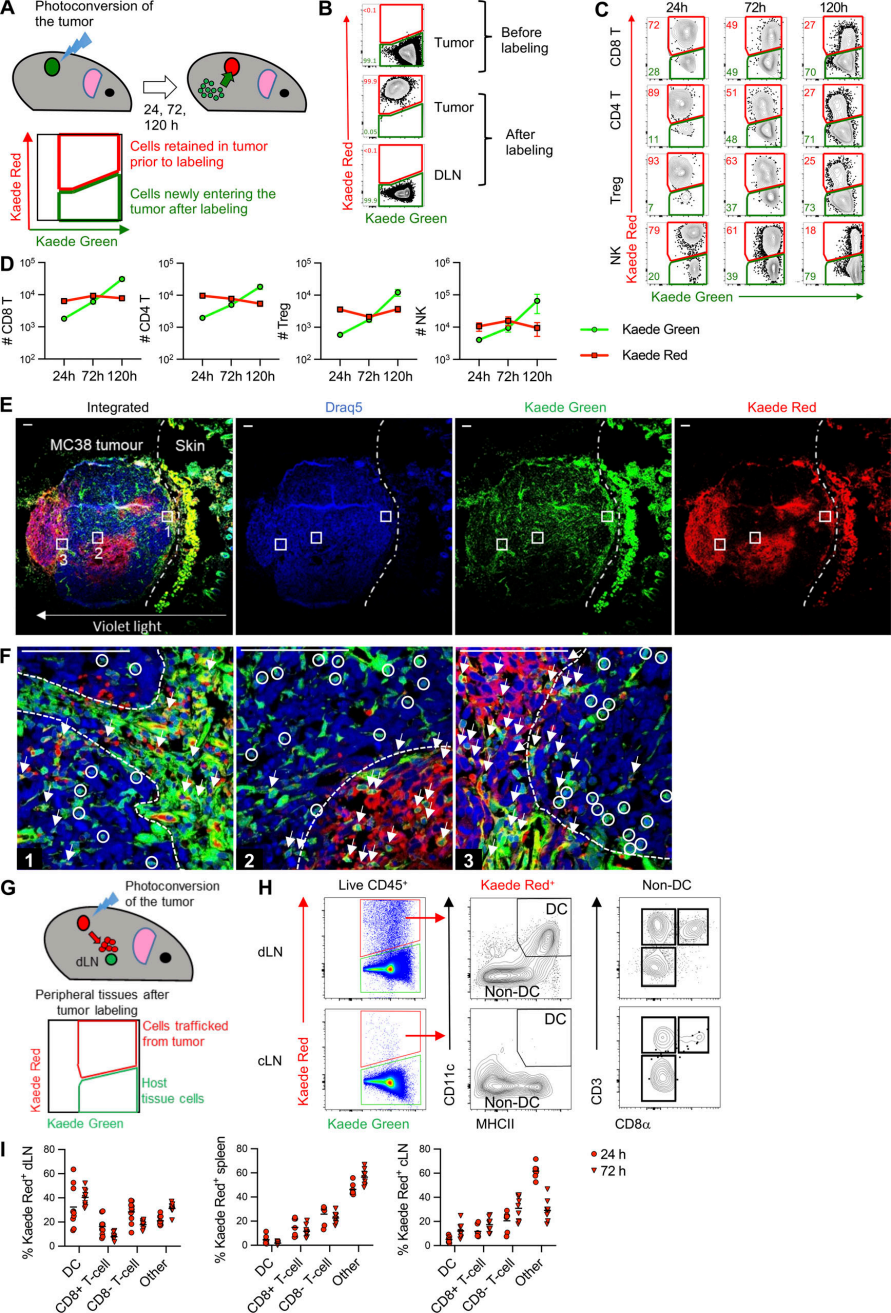
**Defining immune cell entry dynamics in tumors using temporal labeling of the tumor immune compartment. (A)** Cartoon summarizing temporal labeling of the syngeneic cell line MC38 implanted s.c. on the flank of C57BL/6 Kaede mice and photoconverted ∼10 d later by violet light exposure. **(B)** Representative flow cytometry plots showing Kaede Red and Kaede Green expression by CD45^+^ cells isolated from the tumor of non-photoconverted Kaede mice and from the tumor and dLN immediately after photoconversion (0 h), *n* = 6 pooled from two independent experiments. **(C)** Representative flow cytometry plots showing Kaede Red and Kaede Green expression by tumor-infiltrating CD8 T cells (CD11b^−^CD11c^−^CD3^+^CD4^−^CD8^+^), CD4 T cells (CD11b^−^CD11c^−^CD3^+^CD4^+^CD8^−^), Treg (CD11b^−^CD11c^−^CD3^+^CD4^+^CD8^−^Foxp3^+^), and NK cells (CD11b^−/lo^CD11c^−^CD3^−^CD4^−^CD8^−^NK1.1^+^) 24, 72, and 120 h after tumor photoconversion. **(D)** Quantification of Kaede Red^+^ and Kaede Green^+^ CD8 T cells (24 h, *n* = 41; 72 h, *n* = 18; 120 h, *n* = 16), CD4 T cells (24 h, *n* = 39; 72 h, *n* = 17; 120 h, *n* = 16), Tregs (24 h, *n* = 24; 72 h, *n* = 10; 120 h, *n* = 9), and NK cells (24 h, *n* = 9; 72 h, *n* = 5; 120 h, *n* = 3) pooled from six independent experiments. Data presented as mean values ± SEM. **(E and F)** The distribution of Kaede Green^+^ and Kaede Red^+^ cells within MC38 tumors 24 h after photoconversion. **(E)** Images show the tile scan of the whole tumor section with distribution of Kaede Green^+^ cells (also Kaede Red^−^) and Kaede Red^+^ cells (usually also Kaede Green^+^). The DNA-labeling dye Draq5 was used to visualize cell nuclei. Three representative areas as marked by rectangles are shown in higher magnification in F, where circles indicate Kaede Green^+^ cells (also Kaede Red^−^) while arrows indicate Kaede Red^+^ cells (usually also Kaede Green^+^). Dashed line indicates the boundary between tumor cell–enriched areas and Kaede Red^+^ cell–enriched areas. Analysis representative of three tumors from two independent experiments; scale bar represents 100 μm. **(G)** Cartoon summarizing the tracking of Kaede Red^+^ cells from the tumor to the dLN. **(H)** Gating strategy to identify DCs (CD11c^+^MHCII^+^) and T cell subsets in the dLN and cLN. **(I)** Proportion of different immune cell populations with the Kaede Red^+^ hematopoietic cells present within the dLN (24 h, *n* = 10; 72 h, *n* = 10), spleen (24 h, *n* = 7; 72 h, *n* = 10) and cLN (24 h, *n* = 7; 72 h, *n* = 9) at 24 and 72 h after photoconversion pooled from two independent experiments. Bars on scatter plots represent mean values. Values on flow cytometry plots define the percentage of cells within the gate.

We hypothesized that temporal labeling of the entire immune cell compartment of tumors in Kaede photoconvertible mice, as we recently described for LNs (Dutton et al., 2019; Marriott et al., 2017), would enable the characterization of both newly entering and resident TILs. How TILs changed in situ over time could then be determined by sampling these compartments at different times after labeling. To this end, we established syngeneic MC38 tumors subcutaneously on the flank of C57BL/6 Kaede mice and transcutaneously photoconverted the resulting tumor after ∼10 d (Fig. 1 A). Analysis of the hematopoietic compartment immediately after labeling (0 h) confirmed the complete (99.9%) conversion of these cells from the default green fluorescence of the Kaede protein (“Kaede Green^+^”) to the altered Red fluorescent profile (“Kaede Red^+^”, Fig. 1 B). Of note, shielding of the surrounding skin fully protected the dLN from photoconversion (Fig. 1 B). The complete photoconversion of the tumor compartment was dependent upon the size of the tumor, constraining labeling of tumors up to 5–8 mm^2^ in diameter.

**Figure S1.**
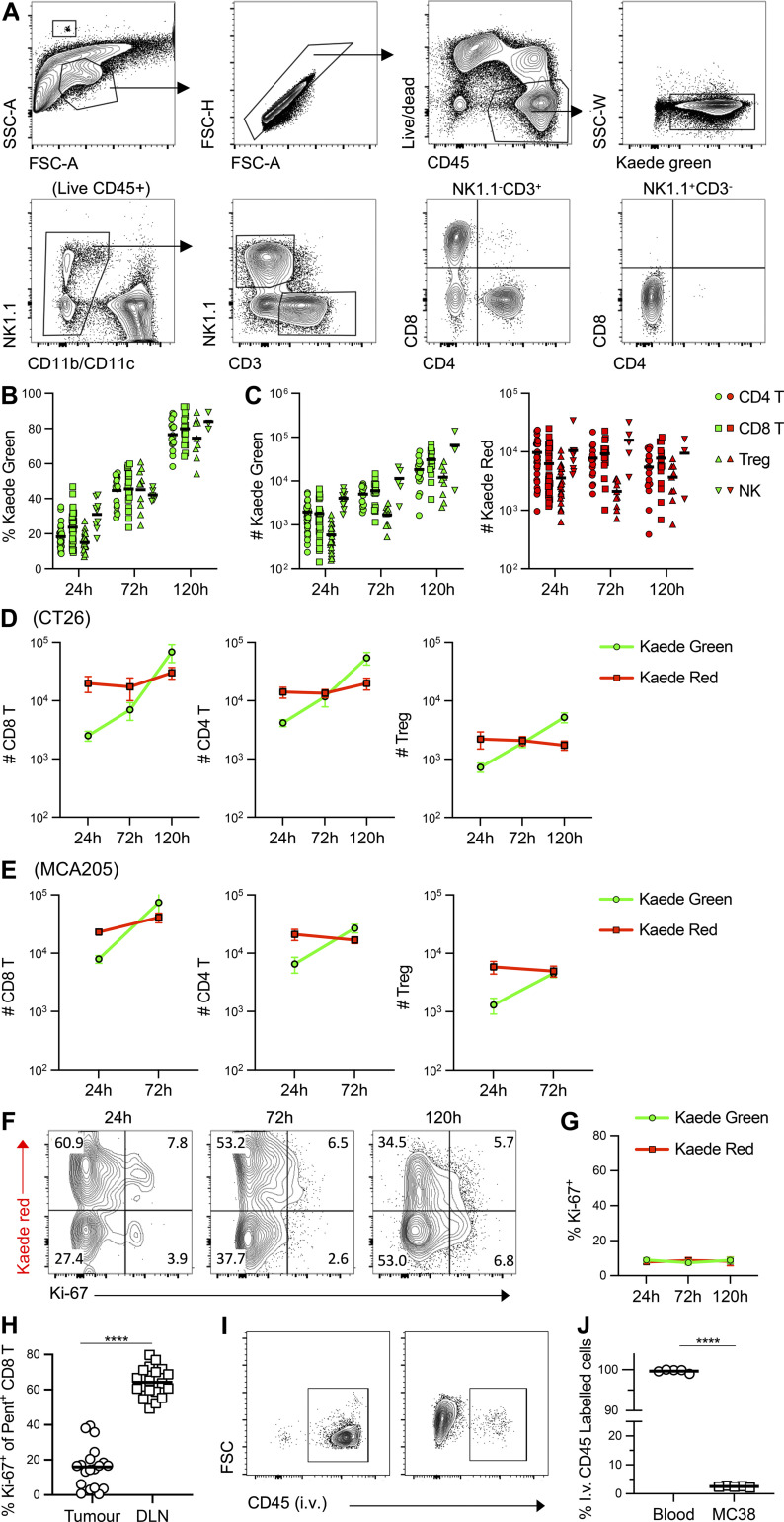
**Limited intratumoral T cell proliferation prevents rapid loss of Kaede Red protein. (A)** Gating strategy for identification of TILs within MC38 tumors. **(B)** Proportion of Kaede Green^+^ CD4 T cells, CD8 T cells, Tregs, and NK cells at 24, 72 and 120 h after photoconversion. **(C)** Total number of Kaede Green^+^ and Kaede Red^+^ CD4 T cells, CD8 T cells, Tregs, and NK cells at 24, 72 and 120 h after photoconversion. For B and C, CD4 T cells: *n* = 39 at 24 h, *n* = 17 at 72 h, *n* = 16 at 120 h; for CD8 T cells: *n* = 41 at 24 h, *n* = 18 at 72 h, *n* = 16 at 120 h; for Tregs: *n* = 24 at 24 h, *n* = 10 at 72 h, *n* = 9 at 120 h; for NK cells: *n* = 9 at 24 h, *n* = 5 at 72 h, *n* = 3 at 120 h) pooled from six independent experiments. Bars represent the median values. **(D)** Quantification of Kaede Red^+^ and Kaede Green^+^ TILs in CT26 tumors over 5 d after photoconversion, CD8 T cells (24 h, *n* = 25; 72 h, *n* = 14; 120 h, *n* = 5), CD4 T cells (24 h, *n* = 25; 72 h, *n* = 13; 120 h, *n* = 5) and Tregs (24 h, *n* = 12; 72 h, *n* = 10; 120 h *n* = 5) pooled from three independent experiments. Data presented as mean values ± SEM. **(E)** Quantification of Kaede Red^+^ and Kaede Green^+^ TILs in MCA205-OVA tumors over 5 d after photoconversion, CD8 T cells (24 h, *n* = 9; 72 h, *n* = 12), CD4 T cells (24 h, *n* = 9; 72 h, *n* = 12) and Tregs (24 h, *n* = 6; 72 h, *n* = 6) pooled from two independent experiments. **(F)** Representative expression of Ki-67 versus Kaede Red in CD8 T cells isolated from MC38 tumors at 24, 72, and 120 h after photoconversion. Values on flow cytometry plots represent percentages. **(G)** Proportion of Kaede Green^+^ and Kaede Red^+^ CD8 T cells expressing Ki-67 over the time course, *n* = 34 at 24 h, *n* = 19 at 72 h, and *n* = 6 at 120 h pooled from six independent experiments. Data was presented as mean values ± SEM. **(H)** Enumeration (%) of Ki-67 expression amongst MC38 neo-Ag specific CD8 T cells in the tumor and dLN (*n* = 19 pooled from five independent experiments). Bars represent mean values. **(I)** Detection of bound CD45 Ab on hematopoietic cells isolated from tumor or blood following i.v. injection. **(J)** Proportion of hematopoietic cells labeled with i.v. administered anti-CD45 Ab in blood and tumor (*n* = 5). Two independent experiments were performed. Data was presented as mean values ± SEM. Statistical significance was tested using paired *t* test (H and J): ****, P ≤ 0.0001.

Having established efficient labeling of all immune cells in the tumor, we analyzed tumors at 24, 72, and 120 h after photoconversion, reasoning that TILs that were only Kaede Green^+^ had entered the tumor after labeling, while Kaede Red^+^ TILs were present within the tumor at the time of photoconversion (Figs. 1 C and S1 A). At 24 h after photoconversion ∼25% of CD8 T cells, CD4 T cells, regulatory T cells (Treg), and natural killer (NK) cells were Kaede Green^+^, and this proportion increased over time such that by 120 h, the majority of all TILs had entered the tumor since labeling (Figs. 1 C and S1 B). The total number of Kaede Green^+^ TILs increased 10-fold over the 5-d timeframe, indicating the continuous and rapid influx of these cells during tumor expansion. In contrast, the total number of Kaede Red^+^ cells appeared relatively constant. Thus the steady drop in the proportion of Kaede Red^+^ cells was explained by the continuous recruitment of new cells into the tumor (Figs. 1 D and S1 C). Comparable recruitment dynamics were observed in CT26 and MCA205 tumor models (Fig. S1, D and E).

Since the intensity of the Kaede Red “label” is reduced through dilution in proliferating cells (Tomura et al., 2008), we assessed Ki-67 expression to investigate whether proliferation might hinder discrimination between newly entering and resident TIL populations. Minimal Ki-67 expression was detected within total or Ag-specific CD8 T cells within the tumor, unlike in the dLN (Fig. S1, F–H), arguing that the proliferation-induced loss of the Kaede Red protein was not a major confounder in our experiments. Using i.v. administration of anti-CD45 Abs prior to culling, we further confirmed that the vast majority of cells isolated from tumor samples had entered the tissue and were not intravascular contaminants (Fig. S1, I and J).

**Figure S2.**
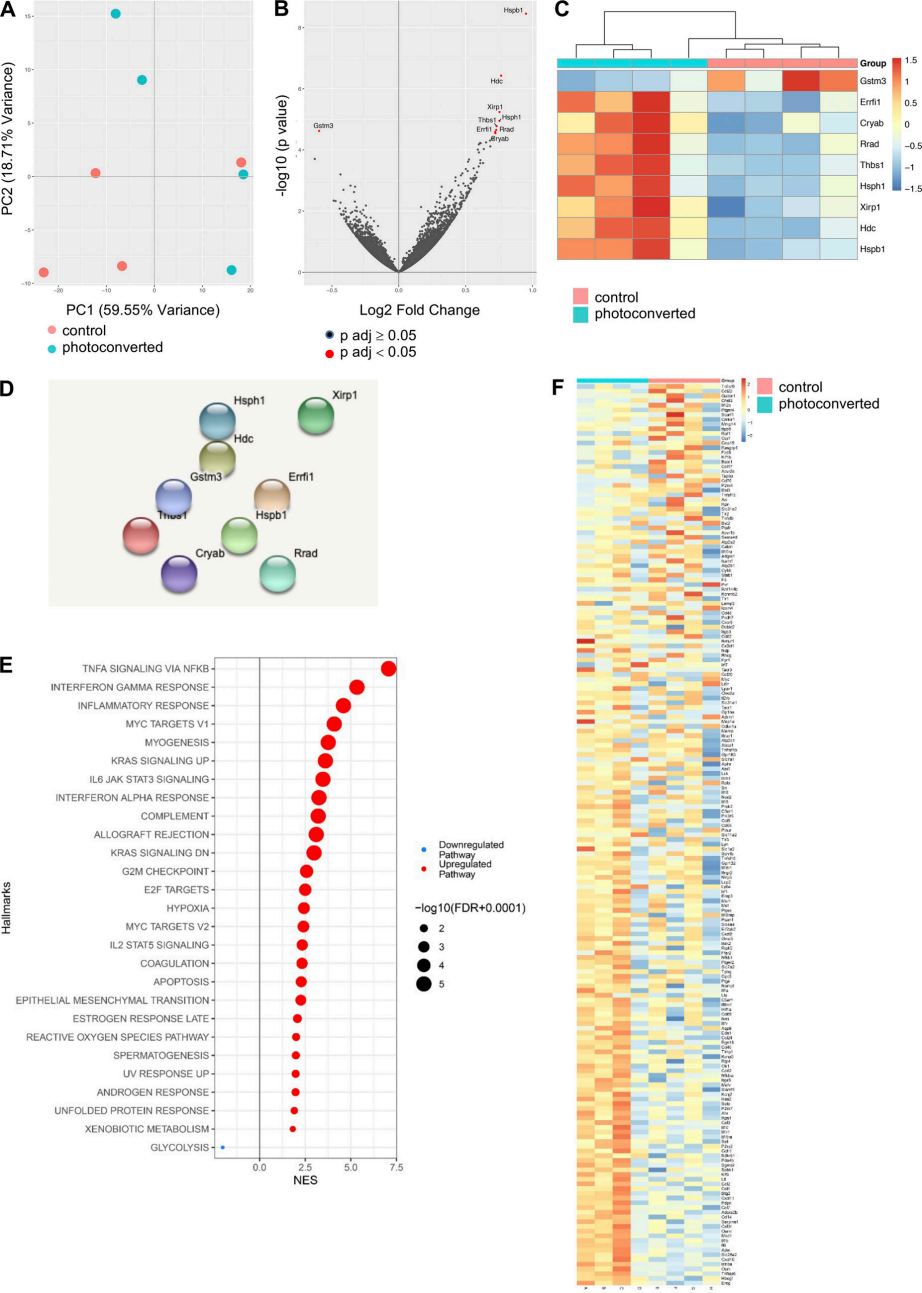
**Photoconversion induces minimal transcriptional changes within the tumor.** To investigate the potential effects of photoconversion upon cells within the tumor, bulk RNA-seq was performed on MC38 tumors 5 h after photoconversion (*n* = 4) alongside non-photoconverted controls (*n* = 4). **(A)** PC analysis. **(B)** Volcano plot with DEGs highlighted in red. **(C)** Heatmap of DEGs. **(D)** STRING analysis of DEGs. **(E)** Gene set enrichment analysis showing upregulated pathways. **(F)** Heatmap of the leading edge genes in pathways identified by gene set enrichment analysis.

To investigate whether the process of photoconversion impacted cells within the tumor, we performed bulk RNA-seq of tumors harvested 5 h after cutaneous light exposure, as well as matched non-photoconverted tumors. Principal components (PC) analysis suggested that the photoconversion state of the tumor influenced PC1, although this effect was variable, with one of the photoconverted tumor samples clustering with the control tumor samples (Fig. S2 A). Only nine genes were significantly differentially expressed between the photoconverted and the non-photoconverted tumors, eight were upregulated and one downregulated (Fig. S2, B and C), and protein–protein interaction analysis using STRING did not identify any functional relationship between the proteins encoded by these nine genes (Fig. S2 D). Gene set enrichment analysis showed that several immune-related/inflammatory gene pathways were upregulated in photoconverted tumors (Fig. S2 E), although none of the leading-edge genes were significantly differentially expressed, reflecting the variable and modest induction of these pathways (Fig. S2 F). Collectively, these data indicate that there was a minimal effect of photoconversion, and the induction of inflammatory pathways resulting from light exposure was limited. We additionally assessed the distribution of the newly entering and resident TILs, which suggested enrichment of Kaede Red^+^ cells within certain regions of the tumor (Fig. 1 E) and confirmed that Kaede Green^+^ and Kaede Red^+^ cells were both distributed throughout the tumor (Fig. 1 F).

Having assessed the extent and rate of immune cell recruitment into the tumor, we further sought to determine the capacity of different immune cells to egress this tissue (Torcellan et al., 2017). Therefore, we analyzed the composition of Kaede Red^+^ cells within the dLN at 24 and 72 h after photoconversion of the tumor (Fig. 1 G). Consistent with their critical role in cross-priming CD8 T cells recognizing tumor neo-Ags (Wculek et al., 2020), the Kaede Red^+^ CD45^+^ population of the dLN, but not the contralateral inguinal LN (cLN) contained a clear CD11c^+^ MHCII^+^ population of DCs (Fig. 1 H). In addition, both CD4^+^ and CD8^+^ T cells were evident amongst the Kaede Red^+^ population within the dLN, but also the cLN and spleen (Fig. 1, H and I), demonstrating that some immune cells egress the tumor and circulate through peripheral lymphoid tissues.

### scRNA-seq of newly entering versus resident TILs

**Figure 2.**
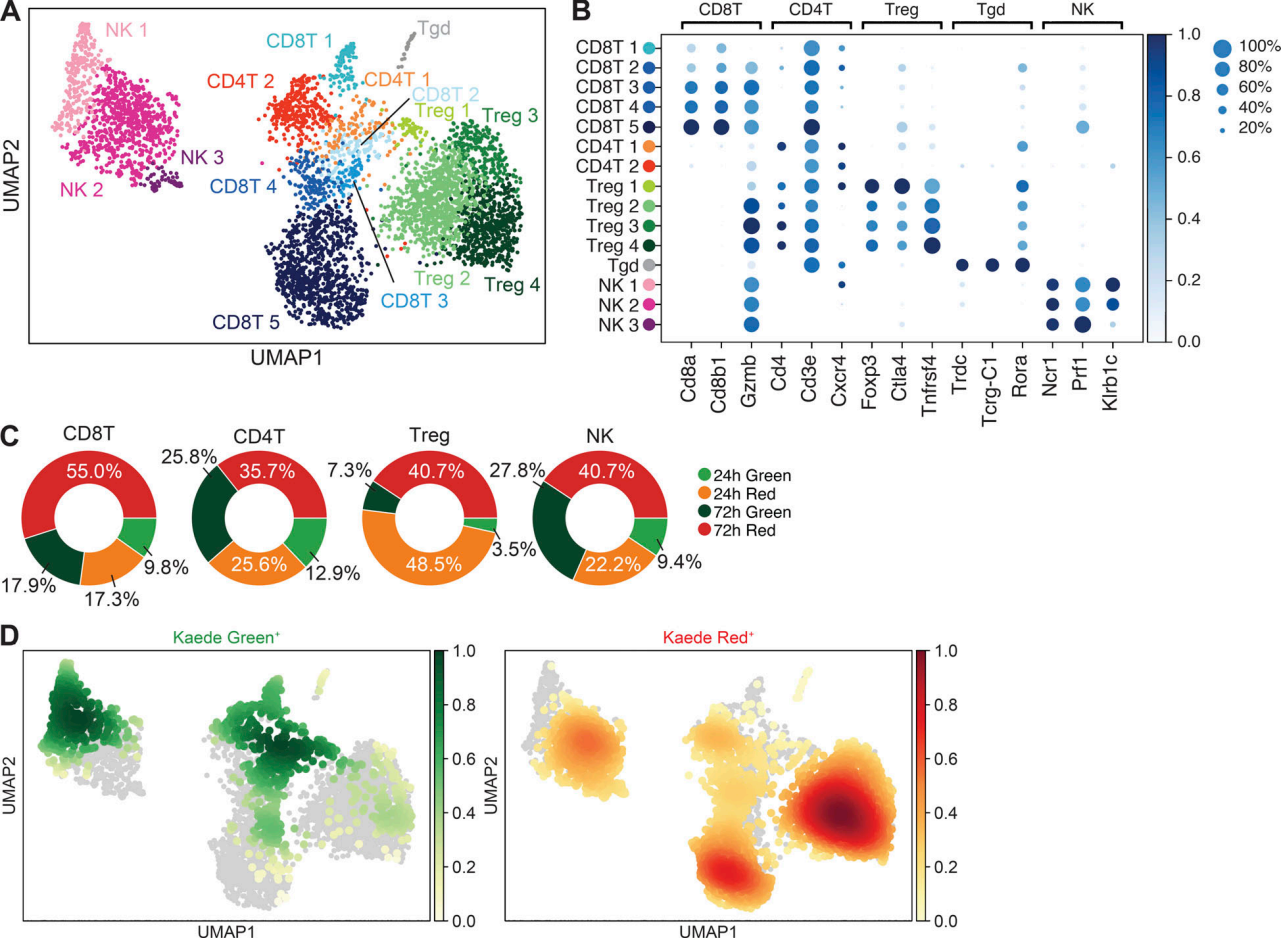
**scRNA-seq analysis of heterogeneity within newly entering and resident TILs.** Kaede Green^+^ and Kaede Red^+^ TILs were isolated by FACS (live CD45^+^Ter119^−^CD11b^−/lo^) from pools of MC38 tumors (*n* = 4 at each time point) at 24 and 72 h after photoconversion, generating four samples (G24, G72, R24, and R72) and analyzed by scRNA-seq. **(A)** UMAP of 4,597 TIL cells colored according to cell clusters (CD8T1-5, CD4T1-2, Treg1-4, NK1-3, and Tgd). **(B)** Mean expression dot plot of canonical marker genes for major cell types. The size of the circles corresponds to the percentage of cells expressing the gene, and the increasing mean expression value (scaled from 0 to 1) corresponds to an increasing gradient from white to blue. **(C)** Sample composition of CD8 T cells, CD4 T cells, Tregs, and NK cells isolated from tumors. **(D)** UMAPs showing the density (gaussian kernel density estimates) of Kaede Green^+^ and Kaede Red^+^ cells in the embedding.

To fully capture cellular heterogeneity amongst the newly entering and tumor-resident TIL populations, we isolated Kaede Green^+^ and Kaede Red^+^ TILs (CD45^+^CD11b^−/lo^) from MC38 tumors at 24 and 72 h after photoconversion (named: G24, G72, R24, R72, respectively) and analyzed their transcriptomes using scRNA-seq. After quality control, unbiased clustering of TILs from all samples revealed 15 distinct clusters, 5 of which were assigned as CD8 T cells, 2 as FoxP3^−^ CD4 T cells, 4 as FoxP3^+^ Tregs, 3 as NK cells, with 1 γδ T cell cluster, based on the expression of canonical marker genes (e.g., *Cd8a*, *CD3e*, *Foxp3*, *Ncr1*; Fig. 2, A and B). Analysis of the cell type–specific clusters confirmed that each contained cells from both time points and included both Kaede Green^+^ and Kaede Red^+^ cells (Fig. 2 C), although Kaede Red^+^ cells dominated as observed in the initial flow cytometric characterization (Fig. 1 C). Notably, the Kaede Green^+^ (G24, G72) and Kaede Red^+^ (R24, R72) cells were not evenly distributed among clusters, but preferentially occupied specific clusters, indicating that newly entering and resident TIL populations have distinct transcriptional profiles (Fig. 2 D). Combined, these data describe a novel in vivo model that can define the phenotype of TILs as they enter the tissue and map transcriptional changes over time within this environment.

### LAG-3 expression defines Tregs retained within the tumor

**Figure S3.**
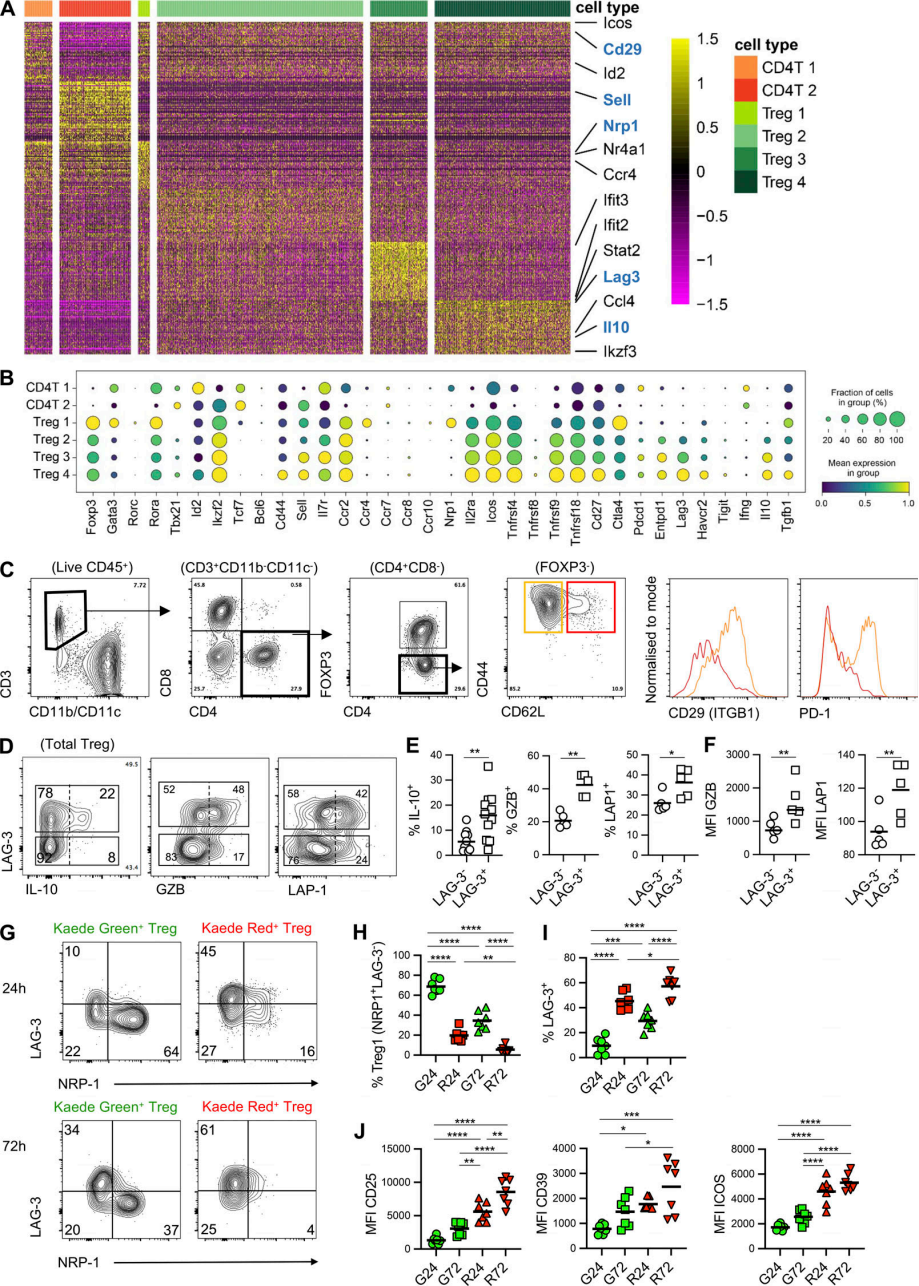
**Differential gene expression amongst CD4 T cell populations within the tumor. (A)** Top 50 DEGs for Treg clusters. **(B)** Mean expression dot plot of selected regulatory T lymphocyte markers. Size of circles correspond to percentage of cells expressing gene and increasing mean expression value (scaled from 0 to 1) corresponds to increasing gradient from purple to blue to green to yellow. **(C)** Representative flow cytometry showing gating strategy to identify two Foxp3^−^ CD4 populations based on CD62L expression and their differential expression of PD-1 and CD29 in MC38 tumors. **(D)** Representative flow cytometry plots showing expression of IL-10, Granzyme B (GZB) and LAP-1 (to detect TGFβ) by LAG-3^+^ and LAG-3^−^ Tregs in MC38 tumors. **(E)** Proportion of LAG-3^+^ and LAG-3^−^ Tregs expressing IL-10 (*n* = 11), GZB (*n* = 5), and LAP-1 (*n* = 5). **(F)** Geometric MFI of GZB and LAP-1 expression by LAG-3^+^ and LAG-3^−^ Tregs (*n* = 5). **(G)** Representative flow cytometry plots showing LAG-3 versus NRP-1 expression by Kaede Green^+^ and Kaede Red^+^ Tregs isolated from CT26 tumors at 24 and 72 h after photoconversion. **(H and I)** Proportion of Kaede Green^+^ and Kaede Red^+^ Tregs in CT26 tumors with (H) the phenotype of “Treg 1” subset (NRP1^+^ LAG-3^−^) and (I) LAG-3^+^ at 24 (*n* = 7) and 72 h (*n* = 7) after photoconversion. **(J)** MFI of CD25, CD39, and ICOS by Kaede Green^+^ and Kaede Red^+^ Tregs in CT26 tumors at 24 and 72 h after photoconversion (*n* = 7). Two independent experiments were performed. Values on flow cytometry plots represent percentages and bars on scatter plots represent the mean values. Statistical significance was tested using paired *t* test (E and F), and ordinary one-way ANOVA with Tukey’s multiple comparisons test (H–J): *, P ≤ 0.05; **, P ≤ 0.01; ***, P ≤ 0.001; ****, P ≤ 0.0001.

Treg-mediated suppression curtails the anti-tumor response (Arce Vargas et al., 2018; Shimizu et al., 1999); however, it is evident that tumor Tregs are heterogeneous in their source and specificity (Hindley et al., 2011; Stockis et al., 2019), limiting our understanding of how the Treg compartment of tumors is formed and sustained. Thus, we initially sought to exploit our new tumor model to define the CD4 T cell compartment within the tumor and then utilize the single-cell transcriptomes to investigate how Tregs change within the tumor microenvironment over time. First, we assessed the top 50 differentially expressed genes (DEGs) alongside the expression of a selected group of genes associated with CD4 T cell and Treg biology (Fig. S3, A and B). The CD4 T cell clusters expressed markers consistent with memory populations including *Cd44*, *Tcf7*, and *Sell*, and were transcriptionally quite distinct from the Treg populations. Flow cytometric analysis of FoxP3^−^ CD4 T cells confirmed the presence of two CD44^+^ populations that could be identified by differential expression of markers such as CD62L, PD-1, and CD29 and likely represented clusters CD4T1/2 (Fig. S3 C).

Focusing on the Treg clusters, it was notable that *Nrp-1* expression, but not *Ikzf2* (Gottschalk et al., 2012; Thornton et al., 2010; Weiss et al., 2012), was only detected within the Treg1 cluster along with *Ccr4*, previously reported to drive Treg recruitment into tumors (Curiel et al., 2004; Ishida and Ueda, 2006). These cells also expressed low levels of genes associated with Treg activation compared with other clusters, including *Il2ra*, *Icos*, *Entpd1*, *Tnfrsf4*, *Tnfrsf9*, and *Tnfrsf18* (Fig. S3 B). In contrast, Treg clusters 2–4 were differentiated by a graded expression of Treg “activation” markers, with Treg4 exhibiting the most suppressive phenotype based upon expression of *Lag3*, *Havcr2*, and *Il10* (Fig. S3 B).

**Figure 3.**
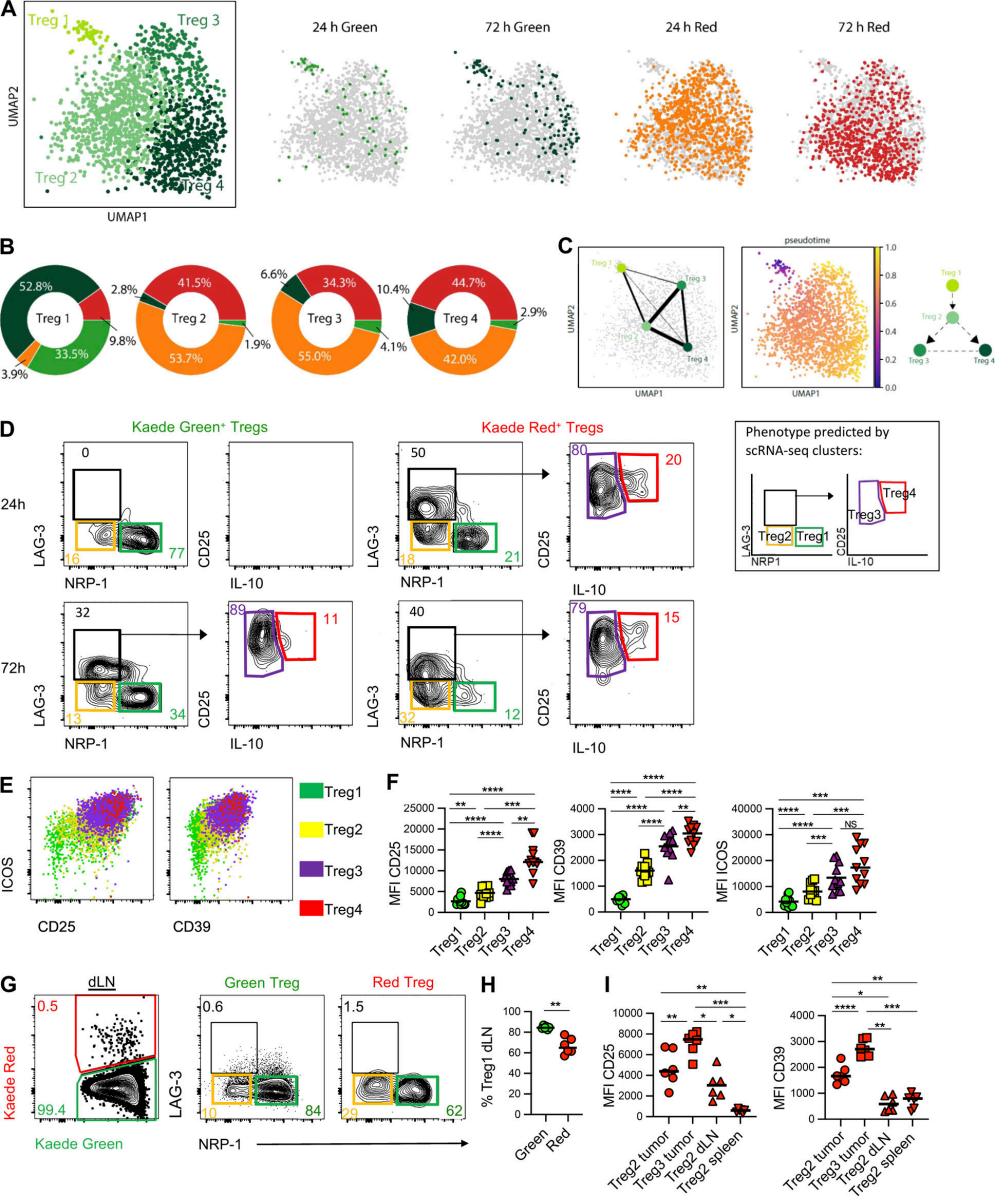
**LAG-3 expression defines Tregs retained within the tumor.** The Treg compartment of MC38 tumors was assessed to define the phenotype of newly recruited and tumor-resident Treg populations. **(A)** UMAPs of 1,749 Tregs colored according to clusters or sample source (G24, G72, R24, and R72). **(B)** Pie charts showing proportions of each sample (G24, G72, R24, R72) in the four Treg clusters. **(C)** UMAPs showing predicted trajectory analysis using PAGA and palantir, rooted in cluster Treg1 alongside pseudotime. Increasing pseudotime value is colored from blue to purple to orange to yellow. **(D)** Representative flow cytometry plots showing Kaede Green^+^ and Kaede Red^+^ Treg populations at 24 and 72 h after photoconversion and defined by differential expression of NRP-1, LAG-3, CD25, and IL-10. **(E)** Representative flow cytometry plots showing the expression of ICOS versus CD25 and ICOS vs. CD39 by Treg1–4, as defined in D. **(F)** Geometric MFI of CD25, ICOS and CD39 by the Treg1–4 populations (*n* = 14). Data were pooled from two time points, i.e., 24 h and 72 h after photoconversion. **(G)** Representative flow cytometry plots defining the Kaede Red^+^ Treg population within the dLN at 24 h after photoconversion and further comparison of NRP-1 versus LAG-3 expression by the Kaede Green^+^ and Kaede Red^+^ Tregs. **(H)** Proportion of Kaede Green^+^ and Kaede Red^+^ Tregs in the dLN with the phenotype of Treg 1 subset (*n* = 6). **(I)** Geometric MFI of CD25 and CD39 expression by Treg2 and Treg 3 phenotype cells in the tumor and LAG-3^−^NRP-1^−^ (Treg2-like) cells in the dLN and spleen (*n* = 6). Two independent experiments were performed. Values on flow cytometry plots define percentage cells within gate. All bars represent median values. Statistical significance was tested in G and J using paired two-tailed *t* test, in H using two-way ANOVA with Sidak’s multiple comparisons test, and in K using one-way ANOVA with Tukey’s multiple comparisons test: *, P ≤ 0.05; **, P ≤ 0.01; ***, P ≤ 0.001; ****, P ≤ 0.0001.

Assessing the distribution of Tregs from the different samples clearly indicated that newly entering Tregs dominated the Treg1 cluster, but were sparsely represented within all other Treg clusters (Fig. 3 A). Indeed, enumerating the proportion of each sample within the clusters confirmed that 85% of the cells in Treg1 were Kaede Green^+^, while >85% of the Tregs in clusters 2–4 came from Kaede Red^+^ samples (Fig. 3 B). Thus, the Treg1 cluster described the majority of newly arrived Tregs in the tumor, while Treg2–4 comprised those retained within the tumor. Pseudotime analysis of the Treg clusters, rooted in Treg1, indicated a cell-fate trajectory that progressed to Treg2 but then bifurcated to give rise to either the Treg3 or Treg4 clusters (Fig. 3 C).

We then sought to validate these transcriptomic differences and establish refined flow cytometry panels that could better distinguish between Treg populations based on time within the tumor. Using the expression of NRP-1, LAG-3, and IL-10 as putative identifiers of the different clusters, this approach confirmed that the vast majority of newly recruited Tregs were NRP-1^+^ LAG-3^−^ (Fig. 3 D) and CD25^+^ ICOS^+^ CD39^−^ (Fig. 3, E and F), consistent with the Treg1 transcriptomic profile. The remaining Kaede Green^+^ Tregs lacked the expression of NRP-1 and LAG-3, but did express CD25, ICOS, and CD39, consistent with the Treg2 transcriptome. Notably, within Kaede Green^+^ Tregs at 72 h after photoconversion, the proportion of NRP-1^+^ LAG-3^−^ cells was reduced and the NRP-1^−^ cells additionally contained a LAG-3^+^ population (Fig. 3 D), suggesting that LAG-3 expression was induced over time in the tumor and potentially identified the Treg3 and Treg4 populations. In support of this, LAG-3^+^ Tregs were evident amongst the retained Kaede Red^+^ cells, while the proportion of NRP1^+^ LAG-3^−^ Tregs was further reduced compared with Kaede Green^+^ Tregs. All LAG-3^+^ Tregs expressed increased levels of CD25, ICOS, and CD39 (Fig. 3, E and F), again consistent with the transcriptomic analysis. Amongst those LAG-3^+^ Tregs with the highest expression of CD25, ICOS, and CD39 were cells capable of producing IL-10 upon restimulation, consistent with the Treg4 cluster. We determined that functionally, LAG-3^+^ Tregs produced significantly more TGFβ, IL-10, and Granzyme B than LAG-3^−^ Tregs (Fig. S3, D–F). We also confirmed that LAG-3 expression identified Tregs retained within tumors in CT26 tumors (Fig. S3, G–J).

Finally, to further understand the fate of intratumoral Tregs, we asked whether Tregs egressed the tumor and if so, whether this was associated with a particular phenotype. Analysis of Kaede Red^+^ Tregs within the dLN after photoconversion of the tumor revealed that the majority were NRP-1^+^ LAG-3^−^ (the “Treg1” phenotype), although a minor NRP-1^−^ LAG-3^−^ population was also detected (Fig. 3, G and H). Thus, either LAG-3^−^ Tregs are not all retained within the tumor or LAG-3 expression is downregulated upon egress. Since Kaede Red^+^ Tregs in the dLN and spleen expressed significantly less CD25 and CD39 than LAG-3^+^ Tregs in the tumor, our data was consistent with the egress of LAG-3^−^ Tregs, rather than simply the downregulation of LAG-3 by Tregs upon or after egress (Fig. 3 I).

Collectively, these data afford new insight into how tumor-infiltrating Treg phenotypes change over time, with LAG-3 expression identifying Tregs that become retained within this environment. Our data further indicate that the expression of NRP-1 is lost over time in the tumor, and this can be at least partially explained by the failure to retain NRP-1^+^ Tregs.

### Rapid establishment of an exhaustion program within tumor-retained CD8 T cells

**Figure 4.**
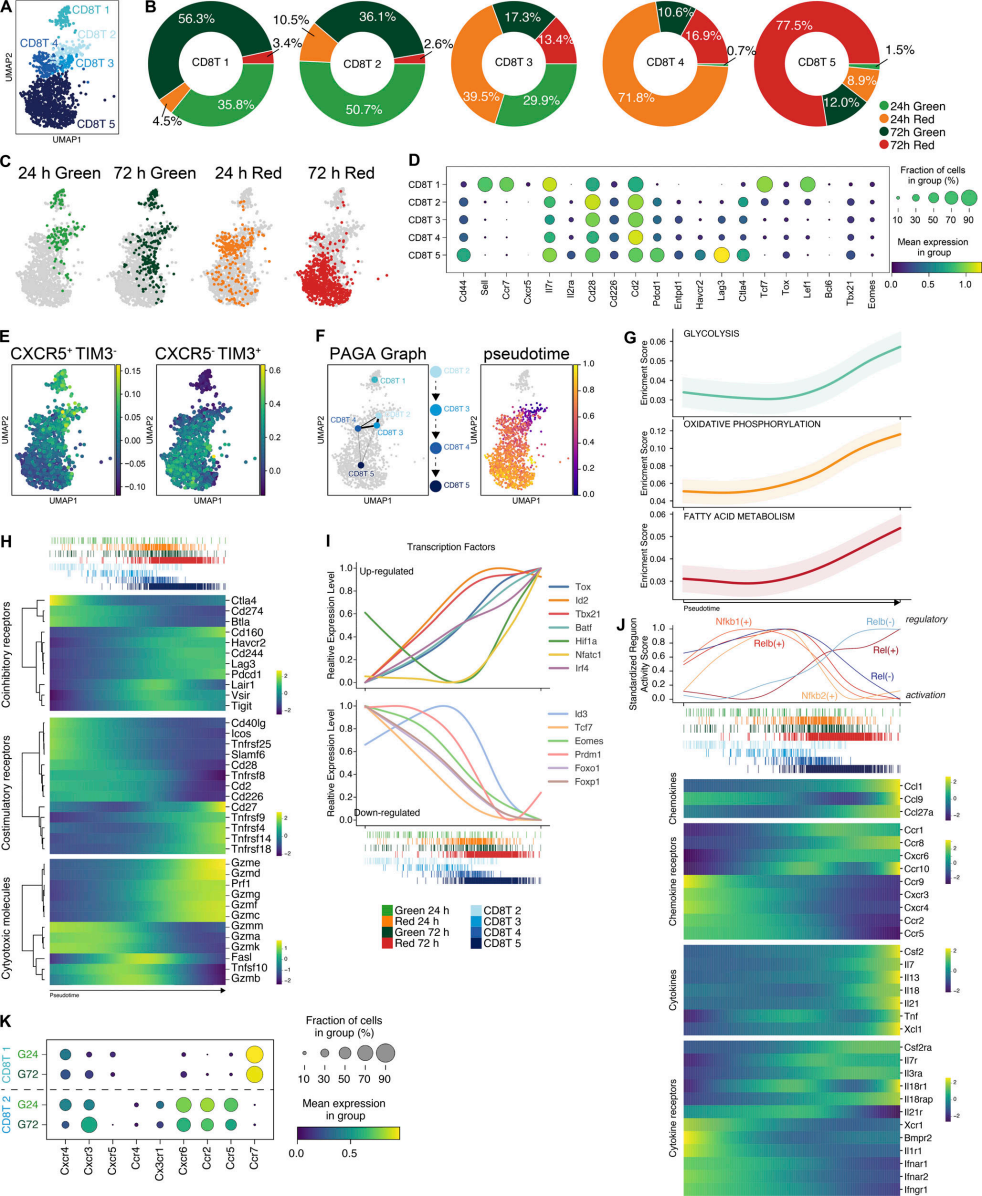
**Rapid establishment of an exhaustion program within tumor-retained CD8 T cells.** Trajectory analyses of the CD8 T cell clusters were used to assess the transcriptomic changes of CD8 T cells after tumor entry and the rate at which an exhausted phenotype was established. **(A)** UMAP of 1,359 CD8 T cells upon tumor entry and retention. **(B)** Pie charts showing proportions of each sample (G24, G72, R24, R72) in the five CD8 T cell clusters. **(C)** CD8 T cell UMAP colored according to sample source. **(D)** Mean expression dot plot of selected genes associated with CD8 T cell phenotype and function. The size of the circles corresponds to the percentage of cells expressing the gene, and the increasing mean expression value (scaled from 0 to 1) corresponds to increasing gradient from purple to blue to green to yellow. **(E)** UMAPs showing a comparison with published CD8 T cell subsets from mice chronically infected with LCMV (Im et al., 2016). **(F)** UMAPs showing predicted trajectory analysis using PAGA and palantir, rooted in cluster CD8T2 alongside pseudotime. Increasing pseudotime value is colored from blue to purple to orange to yellow. **(G)** Gene set enrichment scores for glycolysis, oxidative phosphorylation, and fatty acid metabolism over pseudotime (scaled from 0 to 1). **(H)** Heatmaps showing the expression of coinhibitory, costimulatory, and cytotoxic molecules by CD8 T cells over pseudotime. **(I)** Relative expression of key TFs either up-regulated (top graph) or down-regulated (bottom graph) over pseudotime. **(J)** Top: NF-κB–related TF specificity/activity score plotted as individual line graphs. Bottom: Heatmaps showing the expression of chemokines and cytokines and receptors by CD8 T cells over pseudotime. Expression values/TF specificity/activity scores are binned and averaged at 500 regular intervals. Increasing value on heatmaps corresponds to an increasing gradient from purple to blue to green to yellow. **(K)** Mean expression dot plot of selected chemokine receptors genes (expressed by at least >10% of cells) within Kaede Green^+^ CD8T1 and CD8T2 cells. The size of the circles corresponds to the percentage of cells expressing gene and the color gradient from purple to blue to green to yellow corresponds to increasing mean expression value.

The intratumoral CD8 T cell compartment is diverse, comprised of effector cells becoming increasingly exhausted, alongside naive, memory, stem-like, and bystander CD8 T cell populations (Jansen et al., 2019; Kurtulus et al., 2019; Simoni et al., 2018; Singer et al., 2016). Using our temporal labeling approach, we sought to better define the fate of these different CD8 T cell populations within the tumor over time. Initial clustering of TILs identified five distinct CD8 T cell clusters (Fig. 4 A). The vast majority of the cells within the CD8T1 and CD8T2 clusters were Kaede Green^+^ and had recently entered the tumor, CD8T3 was more evenly split between the different samples, and CD8T4 and CD8T5 contained mostly Kaede Red^+^ cells (Fig. 4, B and C). It was notable that virtually all cells within CD8T5 were from the Kaede Red^+^ 72 h time point, indicating that prolonged residence within the tumor resulted in this transcriptional profile.

**Figure S4.**
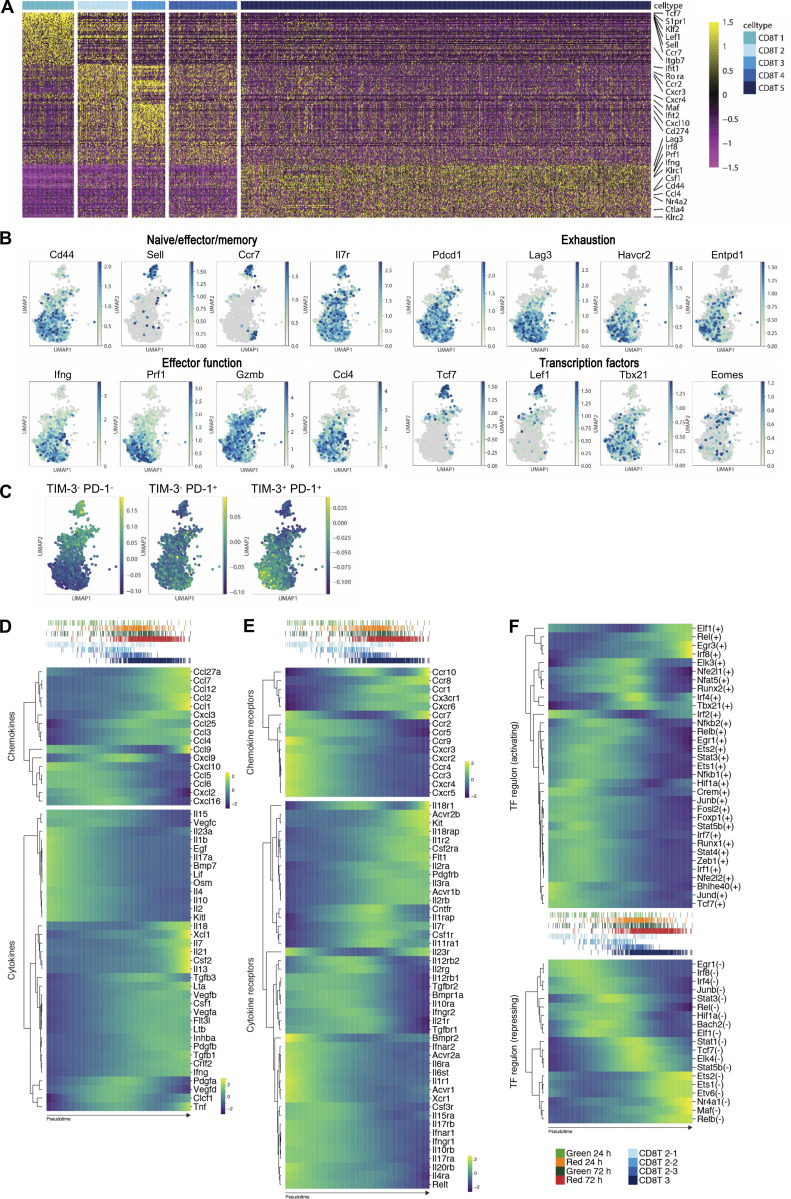
**Expression of key genes within intratumoral CD8 T cells. (A)** Top 50 DEGs for CD8 T cell clusters in MC38 tumors. **(B)** UMAPs showing expression of a selected panel of genes highlighting differentiation stage, exhaustion markers, effector functions and TFs to help inform of CD8 T cell clusters. **(C)** UMAPs showing comparison with published intratumoral CD8 T cell subsets (Singer et al., 2016): Tim-3^−^ PD-1^−^, Tim-3^−^ PD-1^+^, Tim-3^+^ PD-1^+^. **(D)** Heatmaps showing expression of chemokines and cytokines by CD8 T cells over pseudotime. **(E)** Heatmaps showing expression of chemokine receptors and cytokine receptors by CD8 T cells over pseudotime. **(F)** Heatmaps showing predicted activating and repressing TF regulons by CD8 T cells over pseudotime. Expression values/TF specificity/activity scores are binned and averaged to 500 regular intervals and increasing value corresponds to increasing gradient from purple to blue to green to yellow.

To determine the phenotype of the different CD8 T cell clusters, we analyzed the top 50 DEGs (Fig. S4 A) alongside the expression pattern of a selected set of genes, including the homing receptors used to define naive and memory subsets (Sallusto et al., 1999), exhaustion markers, effector functions, and key transcription factors (TFs) associated with different CD8 T cell states (Figs. 4 D and S4 B). Considering the phenotype of CD8 T cells that had newly entered the tumor, the abundance of TF transcripts such as *Tcf7*, *Klf2*, and *Lef1*, as well as homing molecules such as *Sell* and *Ccr7*, in the absence of inhibitory receptor expression (e.g., *Pdcd1*) and with low levels of *Cd44*, indicated that CD8T1 likely contained naive CD8 T cells. Since cells within the CD8T2 cluster co-expressed *Tcf7*, *Cd44*, *Il7r*, and *Pdcd1*, we anticipated that memory and stem-like CD8 T cell populations were likely present here. The expression of canonical markers of activation and effector function (*Cd44*, *Ifng*, *Prf1*, *Gzmb*, and *Ccl4*), the TFs driving this program (*Tbx21* and *Eomes*), and the inhibitory receptors (including *Pdcd1*, *Lag3*, and *Havcr2*) suggested that CD8T4 represented activated effector cells becoming exhausted in CD8T5. To explore this further, we compared these data with published datasets defining the transcriptome of intratumoral CD8 T cells. The differential expressions of PD-1 and TIM-3 have been used to define three distinct populations within MC38 tumors (Kurtulus et al., 2019; Singer et al., 2016), including a PD-1^−^ TIM-3^−^ subset thought to comprise of naive, memory, and stem-like cells, through to exhausted PD-1^+^ TIM-3^+^ cells. Gene set enrichment analysis indicated an enrichment of the PD-1^−^ TIM-3^−^ signature in CD8T1 and 2, whilst the PD-1^+^ TIM-3^+^ “exhaustion” signature was enriched in CD8T5 (Fig. S4 C). Similarly, the signature of CXCR5^+^ TIM-3^−^ stem-like CD8 T cells identified during chronic LCMV infection (Im et al., 2016) was also enriched within CD8T1 and CD8T2 (Fig. 4 E).

To precisely define the transcriptional changes occurring in CD8 T cells after tumor entry, we applied a pseudotime analysis to the CD8 T cell clusters. Ignoring the putative naive population (CD8T1), this indicated a cell fate trajectory that progressed from CD8T2 → CD8T3 → CD8T4 → CD8T5 (Fig. 4 F). The analysis of metabolomic signatures over pseudotime indicated profound changes in glycolysis, oxidative phosphorylation, and fatty acid metabolism as CD8 T cells adapted to the tumor microenvironment (Fig. 4 G). We observed the expected increase in the expression of inhibitory receptors over pseudotime, including *Pdcd1*, *Lag3*, *Cd244*, *Havcr2*, *Cd160*, and *Tigit* as the CD8 T cells acquired an exhausted transcriptomic signature (Fig. 4 H); however, the expression of *Cd274* (encoding PD-L1) and *Btla* expression appeared to decrease (Haymaker et al., 2012; Wherry et al., 2007). There was a general reduction in costimulatory receptor expression, including *Cd28*, *Icos*, and *Cd2*; however, some TNF receptor super family members such as *Cd27*, *Tnfrsf4*, and *Tnfrsf9* progressively increased (Fig. 4 H). There were further striking changes in the expression of cytotoxic molecules (e.g., switch from *Gzma*, *Gzmk* to *Gzmc-f*; Fig. 4 H), and chemokines (e.g., upregulation *Ccl1*, *Ccl2*, *Ccl7*, and *Ccl27*) associated with worse cancer prognosis (Eckstein et al., 2020; Hwang et al., 2012; Kuehnemuth et al., 2018; Simonetti et al., 2006; Fig. S4 D). The expression of *Il2* was rapidly downregulated as previously described for exhausted CD8 T cells (Wherry et al., 2007); however, *Ifng* expression appeared to be maintained (Williams et al., 2017). The analysis of chemokine receptors (Fig. S4 E) highlighted a complete switch in expression from molecules mediating recruitment (*Ccr2*, *Ccr3*, *Ccr4*, *Ccr5*, *Cxcr3*, *Cxcr4*, *Cxcr5*; Harlin et al., 2009; Nagarsheth et al., 2017) to receptors that potentially contribute to retention within the tumor (Ccr8, Ccr10; Facciabene et al., 2011; Plitas et al., 2016).

To investigate how these transcriptomic changes were orchestrated, we assessed TF expression across pseudotime, revealing the increased expression of key TFs associated with establishing the exhausted program, including *Batf*, *Id2*, *Irf4*, *Tbx21*, and *Tox* (Fig. 4 I). The upregulation of *Hif1a* and *Nfatc1*, also associated with T cell exhaustion, was notably delayed in comparison, while a gradually reduced expression of *Tcf7*, *Eomes*, *Prdm1*, *Foxo1*, and *Foxp1* was observed (Fig. 4 I). Since TF activity may not be accurately reflected by the transcript level per se, we performed TF regulon enrichment analysis among genes that were significantly differentially expressed along pseudotime (Fig. S4 F). This identified 33 activating regulons and 19 repressing regulons predicted to regulate the expression of these genes. Notable among these regulons were Rel family TFs, including *Nfkb1*, *Nfkb2*, *Rel*, and *Relb.* We observed distinct patterns of *Rel* family regulon activity across pseudotime (Fig. 4 J), and genes predicted to be controlled by these TFs included chemokines (*Ccl1*, *Ccl9*) and chemokine receptors (*Ccr9*, *Ccr10*) that showed marked variation over pseudotime. Our analysis implicates the *Rel* TFs as key orchestrators of on-going localization of CD8 T cells within the tumor.

Finally, we specifically compared chemokine receptor expression within the newly entering CD8T1 and CD8T2 cells to potentially identify molecular interactions involved in the recruitment of TCF1^+^ PD-1^+^ CD8 T cells into the tumor. Elevated expression of multiple receptors in CD8T2, including *Cxcr6*, *Ccr2*, and *Ccr5*, suggested several candidate pathways (Fig. 4 K). Combined, these data provide a distinct characterization of the transcriptomic changes within CD8 T cells over time within the tumor microenvironment.

### Tracking Ag-specific CD8 T cells over time to validate transcriptomic changes

**Figure 5.**
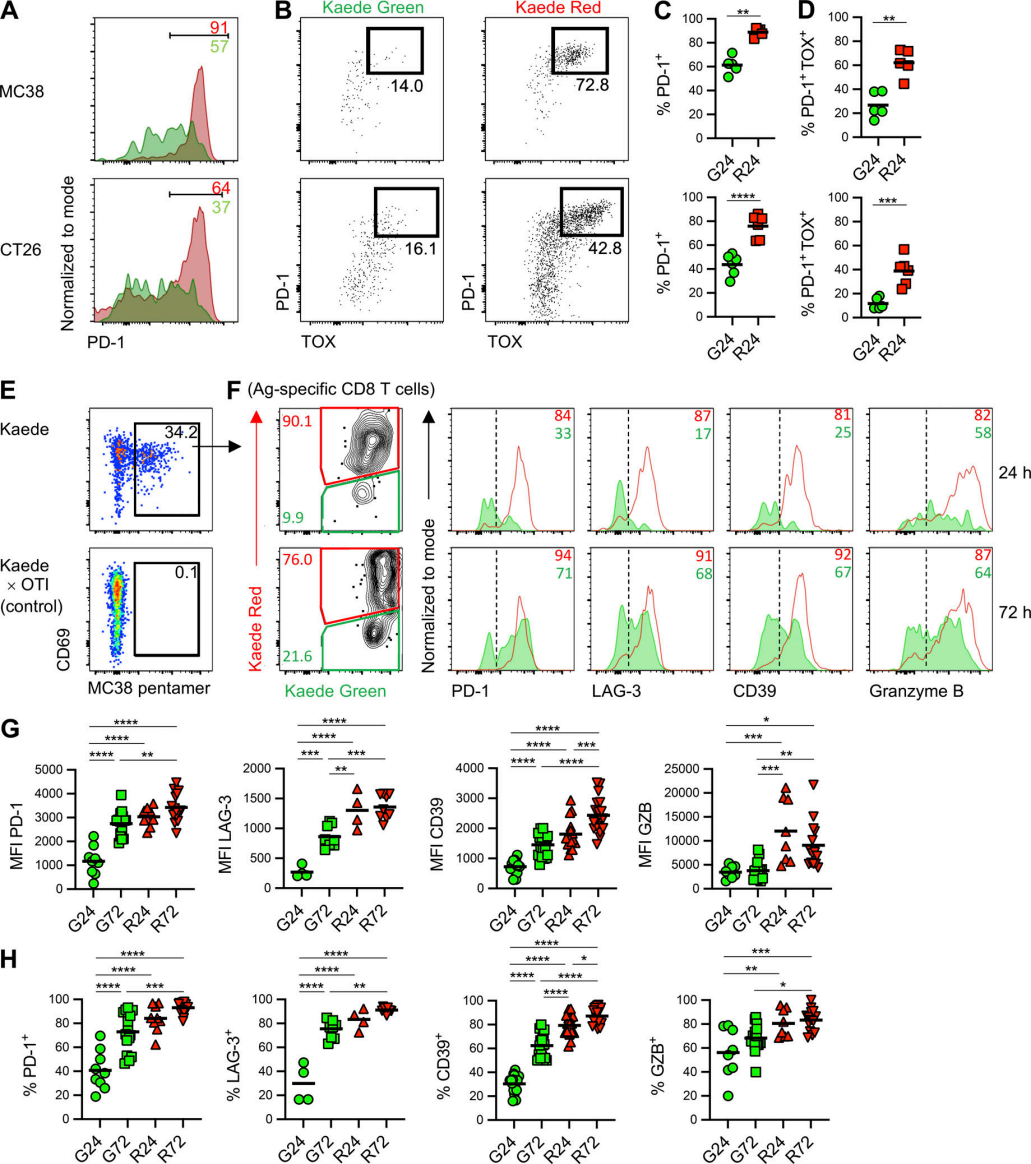
**Tracking Ag-specific CD8 T cells over time to validate transcriptomic changes. (A)** Representative histograms showing PD-1 expression by Kaede Green^+^ and Kaede Red^+^ CD8 T cells (CD44^+^CD62L^−^) 24 h after photoconversion of MC38 (*n* = 5) and CT26 tumors (*n* = 6). **(B)** Representative flow cytometry plots showing expression of PD-1 versus TOX by Kaede Green^+^ and Kaede Red^+^ CD8 T cells (CD44^+^CD62L^−^) 24 h after photoconversion of MC38 (*n* = 5) and CT26 tumors (*n* = 6). **(C and D)** The proportion of (C) PD-1^+^ and (D) PD-1^+^TOX^+^ CD8 T cells (CD44^+^CD62L^−^) amongst Kaede Green^+^ and Kaede Red^+^ CD8 T cells 24 h after photoconversion of MC38 (*n* = 5) and CT26 tumors (*n* = 6). Two independent experiments were performed. **(E)** Identification of MC38 neo-Ag specific CD8 T cells in MC38 tumors in Kaede mice versus OTI × Kaede controls. **(F)** Representative flow cytometry plots and histograms phenotyping Kaede Green^+^ and Kaede Red^+^ MC38 neo-Ag specific CD8 T cells isolated from MC38 tumor at 24 and 72 h after photoconversion. **(G and H)** Geometric MFI (G) and proportion (H) of T cells in F assessed for expression of PD-1 (*n* = 9 at 24 h and *n* = 16 at 72 h), LAG-3 (*n* = 4 at 24 h and *n* = 9 at 72 h), CD39 (*n* = 16 at 24 h and *n* = 18 at 72 h) and Granzyme B (*n* = 8 at 24 h and *n* = 14 at 72 h). Data were pooled form four independent experiments. Values on flow cytometry plots define percentage of cells within gate. Bars on scatter plots represent the median values. Statistical significance was tested using ordinary one-way ANOVA with Tukey’s multiple comparisons test: *, P ≤ 0.05; **, P ≤ 0.01; ***, P ≤ 0.001; ****, P ≤ 0.0001.

To validate the key transcriptional differences that defined the CD8 T cell clusters in our scRNA-seq analysis, we used flow cytometry to track changes in protein expression over time. In both MC38 and CT26 tumors, the majority of CD8 T cells retained within the tumor for at least 24 h (Kaede Red^+^) expressed high levels of PD-1, in contrast to those newly entering (Kaede Green^+^), and furthermore, only these Kaede Red^+^ cells contained a clear PD-1^+^ TOX^+^ population (Fig. 5, A–D). To specifically assess changes in Ag-specific CD8 T cells over time, we tracked an endogenous CD8 T cell response to the MC38 neo-Ag (KSPWFTTL) using MHCI pentamers (Fig. 5 E). Ag-specific CD8 T cells newly recruited into the tumor expressed minimal levels of PD-1, LAG-3, and CD39 alongside reduced amounts of Granzyme B compared with the retained populations (Fig. 5 F). A stepwise increase in the expression of all of these molecules was observed over time, such that CD8 T cells retained within the tumor for at least 72 h robustly expressed PD-1, LAG-3, CD39, and the highest level of Granzyme B (Fig. 5, G and H). Combined with the transcriptional analysis, these data indicate that all CD8 T cells retained within the tumor for at least 3 d developed an exhausted phenotype.

### TCF-1^+^ PD-1^+^ CD8 T cells continuously traffic through tumors

**Figure 6.**
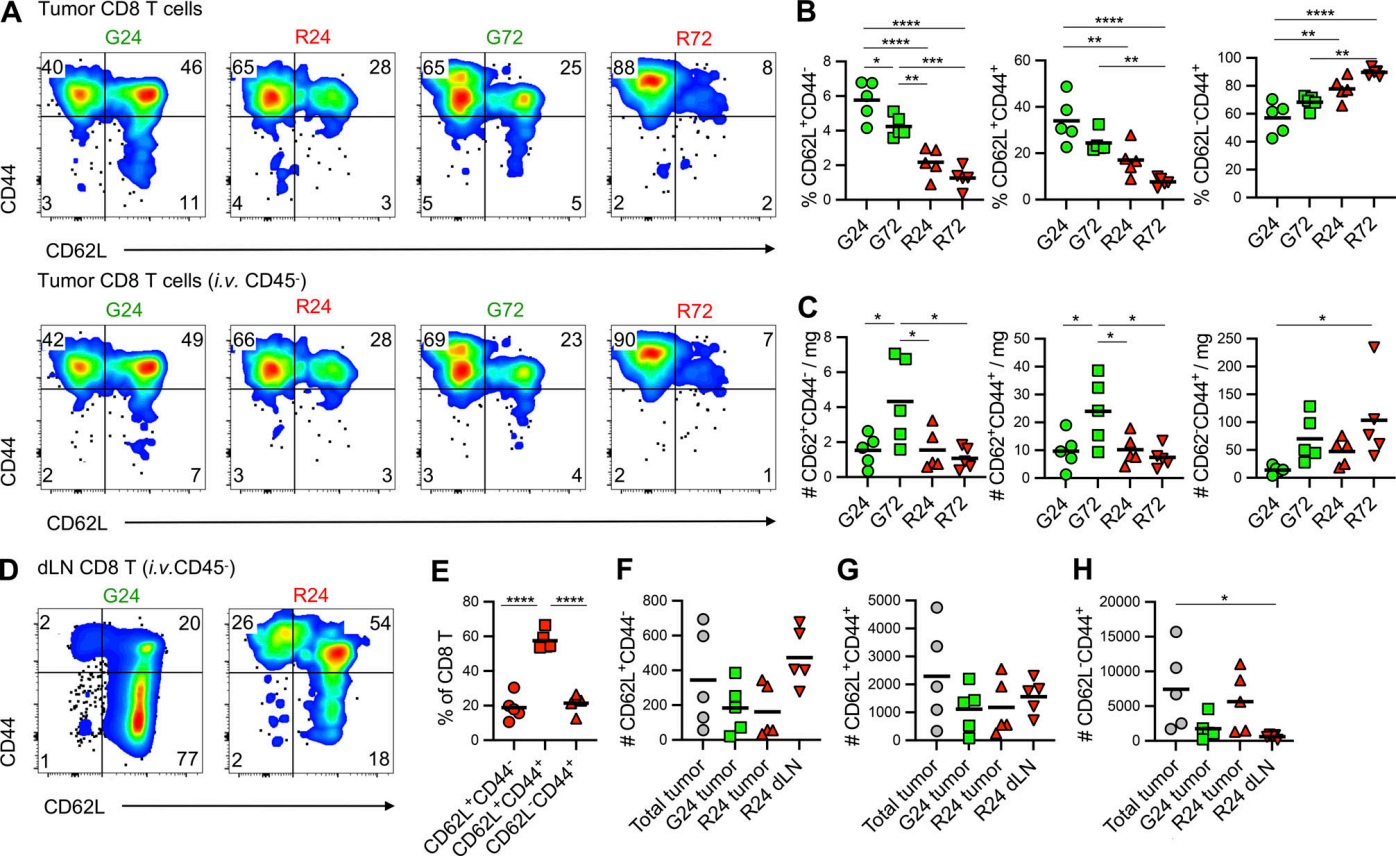
**Naive and Tcm CD8 T cell populations egress the tumor to the dLN.** The CD8 T cell compartment of MC38-Ova tumors was assessed 24 and 72 h after photoconversion on day 11. **(A)** Representative flow cytometry plots showing CD44 vs. CD62L expression by CD8 T cells (±gating on i.v. anti-CD45 labeling shown in upper and lower panels). **(B)** Proportion of naive (CD62L^+^ CD44^−^), Tcm (CD62L^+^ CD44^+^) and effector (CD62L^−^ CD44^+^) cells within the CD8 population (i.v. CD45^−^) in the Kaede Green^+^ and Kaede Red^+^ samples. **(C)** Number of naive (CD62L^+^CD44^−^), Tcm (CD62L^+^ CD44^+^) and effector (CD62L^−^ CD44^+^) cells per mg tumor. **(D)** Representative flow cytometry plots showing CD44 vs. CD62L expression by Kaede Green^+^ and Kaede Red^+^ CD8 T cells within the dLN, 24 h after tumor photoconversion. **(E)** The proportion of the Kaede Red^+^ CD8 T cell population in the dLN with naive, Tcm, or effector phenotype. **(F–H)** The number of (F) naive (CD62L^+^ CD44^−^), (G) Tcm (CD62L^+^ CD44^+^) and (H) effector (CD62L^−^ CD44^+^) cells within the dLN, compared to the total, Kaede Green^+^ and Kaede Red^+^ tumor populations. *n* = 5 at each time point. Two independent experiments were performed. Values on flow cytometry plots represent percentages and bars on scatter plots represent the mean values. Statistical significance was tested using Ordinary one-way ANOVA with Tukey’s multiple comparisons test: *, P ≤ 0.05; **, P ≤ 0.01; ***, P ≤ 0.001; ****, P ≤ 0.0001.

Our temporal labeling had determined that TCF-1 expressing CD8 T cells were abundant amongst the CD8 T cell populations entering the tumor; however these transcriptional profiles were not retained within the tumor over time, consistent with previous studies that indicated the loss of TCF-1 expression (Philip et al., 2017). To initially investigate the fate of different CD8 T cell populations after tumor entry, we assessed the expression of CD44 vs. CD62L amongst Kaede Green^+^ and Kaede Red^+^ CD8 T cells at 24 and 72 h after photoconversion. From this simple analysis, distinct naive (CD44^−^ CD62L^+^ and central memory [Tcm; CD44^+^ CD62L^+^]) populations were evident, particularly amongst the newly entering CD8 T cells, alongside a CD44^+^ CD62L^−^ population that would include effector and exhausted populations (Fig. 6 A). The proportion and number of both CD44^−^ CD62L^+^ naive and CD44^+^ CD62L^+^ Tcm CD8 T cells decreased over time in the tumor (Fig. 6, B and C). Furthermore, it was evident that a substantial proportion of the naive T cell population was present within the tumor vasculature rather than actually penetrating the tumor tissue, likely resulting in the overrepresentation of this cell type within our transcriptomic analysis. We then asked whether egress from the tumor via lymphatic drainage might explain the loss of these populations over time. Analysis of the Kaede Red^+^ CD8 T cells within the dLN 24 h photoconversion revealed clear naive and Tcm populations that comprised ∼75% of the CD8 T cells egressing the tumor (Fig. 6, D and E). Notably, comparable numbers of naive and Tcm cells were present within the Kaede Red^+^ fraction in the dLN and the tumor (Fig. 6, F–H). While the loss of CD62L expression from intratumoral CD8 T cells may impair our ability to detect these TCF-1^+^ populations by flow cytometry, collectively, our transcriptomic and cellular analysis suggest that many TCF-1^+^ CD8 T cells are not retained in the tumor, rather they egress the tissue to return to the periphery.

**Figure 7.**
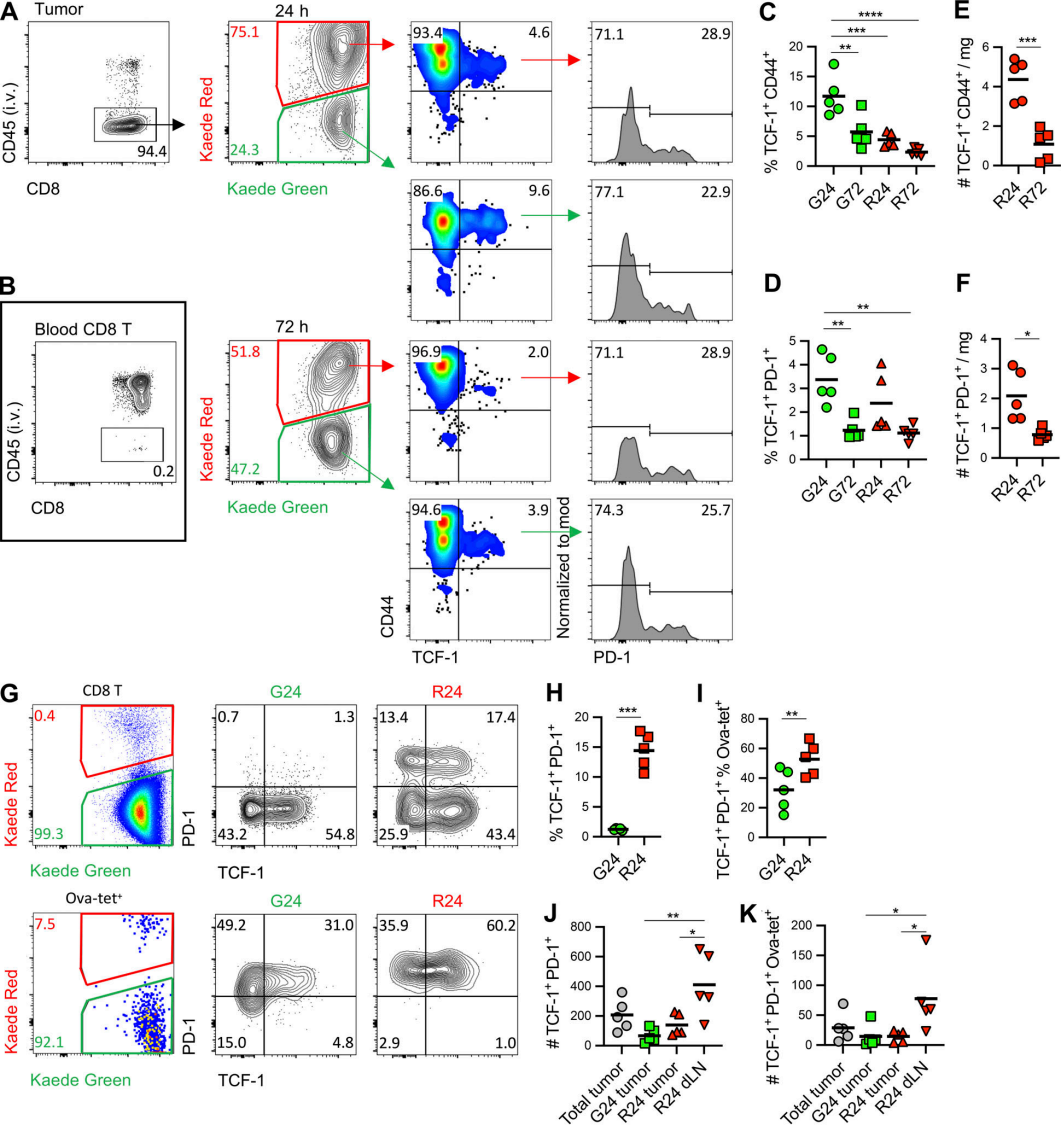
**TCF-1**^**+**^ **PD-1**^**+**^ **CD8 T cells continuously traffic through tumors.** The recruitment and egress of intratumoral TCF-1^+^ PD-1^+^ CD8 T cells was assessed in MC38-Ova bearing Kaede hosts using flow cytometry. Intravenous administration of anti-CD45 Abs was used to discriminate between cells within the tumor or the tumor vasculature. **(A)** Representative flow cytometry plots identifying TCF-1^+^ PD-1^+^ CD8 T cells within MC38-Ova tumors at 24 and 72 h after photoconversion. **(B)** Analysis of labeling of CD8 T cells within the blood after i.v. anti-CD45 Ab administration. **(C and D)** Proportion of TCF-1^+^ CD44^+^ (C) and TCF-1^+^ PD-1^+^ (D) amongst the Kaede Green^+^ and Kaede Red^+^ CD8 T cell compartment isolated from MC38 tumors at 24 (*n* = 5) and 72 h (*n* = 5) after photoconversion. **(E and F)** Number per mg tumor of Kaede Green^+^ and Kaede Red^+^ TCF-1^+^ CD44^+^ (E) and TCF-1^+^ PD-1^+^ (F) population isolated from MC38 tumors weight at 24 (*n* = 5) and 72 h (*n* = 5) after photoconversion. **(G)** Representative flow cytometry plots defining TCF-1^+^ PD-1^+^ population amongst Kaede Green^+^ and Kaede Red^+^ CD8 T cells or Ova-specific T cells (Ova-tet^+^) within the dLN at 24 and 72 h after photoconversion. **(H and I)** Proportion of TCF-1^+^ PD-1^+^ population amongst Kaede Green^+^ and Kaede Red^+^ CD8 T cells (H) or Ova-specific T cells (I) within the dLN at 24 h after photoconversion (*n* = 5). **(J and K)** Number of TCF-1^+^ PD-1^+^ CD8 T cells (J) or Ova-specific T cells (K) in the tumor and dLN at 24 h after photoconversion (*n* = 5). Two independent experiments were performed. Values on flow cytometry plots represent percentages and bars on scatter plots represent the median values. Statistical significance was tested using ordinary one-way ANOVA with Tukey’s multiple comparisons tests: *, P ≤ 0.05; **, P ≤ 0.01; ***, P ≤ 0.001; ****, P ≤ 0.0001.

Amongst the intratumoral TCF-1^+^ CD8 T cells, it is the PD-1^+^ “stem-like” population that is thought to sustain the effector response (Siddiqui et al., 2019). Furthermore, these cells are proposed to form resident populations in human tumors (Jansen et al., 2019). However, the lack of Kaede Red^+^ cells contributing to the CD8T2 cluster indicated that these cells were not retained within MC38 tumors over time. Therefore, we tracked TCF-1^+^ PD-1^+^ CD8 T cells by flow cytometry, again using i.v. CD45 Ab labeling to distinguish cells within the tumor vasculature (Fig. 7, A and B). The proportion of both TCF-1^+^ and TCF-1^+^ PD-1^+^ cells within the Kaede Green^+^ and Kaede Red^+^ CD8 T cell populations decreased over time (Fig. 7, C and D), which equated to a significant loss in the number of Kaede Red^+^ TCF-1^+^ and TCF-1^+^ PD-1^+^ CD8 tumor T cells between 24 and 72 h (Fig. 7, E and F). These data indicate that the TCF-1^+^ PD-1^+^ population of tumors must be constantly replenished with newly entering cells, an observation consistent with recent studies (Connolly et al., 2021). The loss of the TCF-1^+^ PD-1^+^ population over time could simply reflect their differentiation into effector populations, visualized transcriptomically as clusters CD8T3–5. However, this would appear to be in the absence of self-renewal. TCF-1^+^ PD-1^+^ cells appear to favor lymphoid tissue environments (Abdelsamed et al., 2020; Im et al., 2020); thus we hypothesized that the TCF-1^+^ PD-1^+^ population might be preserved by egressing the tumor. To test this, we analyzed Kaede Red^+^ CD8 T cells within the dLN 24 h after photoconversion and indeed observed a distinct TCF-1^+^ PD-1^+^ population that accounted for ∼15% of the total CD8 T cells that had left the tumor (Fig. 7, G and H). More strikingly, within the Ova-specific population, TCF-1^+^ PD-1^+^ cells formed ∼50% of the population (Fig. 7 I). Furthermore, the total number of TCF-1^+^ PD-1^+^ CD8 T cells present within the Kaede Red^+^ fraction of the dLN was greater than the number retained within the tumor (Fig. 7, J and K), further supporting the notion that the loss of this population from the tumor is at least partially the result of lymphatic egress.

Collectively, these data highlight that while effector CD8 T cells responding to tumor Ags become retained within the tumor and rapidly develop an exhausted phenotype due to chronic Ag exposure, the TCF-1–expressing populations do not form stable tumor-resident populations and rather, are continuously trafficking between the tumor and the periphery. For the TCF-1^+^ PD-1^+^ population, this means that the intratumoral niche is dynamic, constantly replenished by newly recruited cells which balance the differentiation to effector cells and egress to lymphoid tissue via lymphatic drainage.

### Blockade of PD-L1 sustains effector function in newly entering TILs as well as reinvigorating exhausted T cells

**Figure 8.**
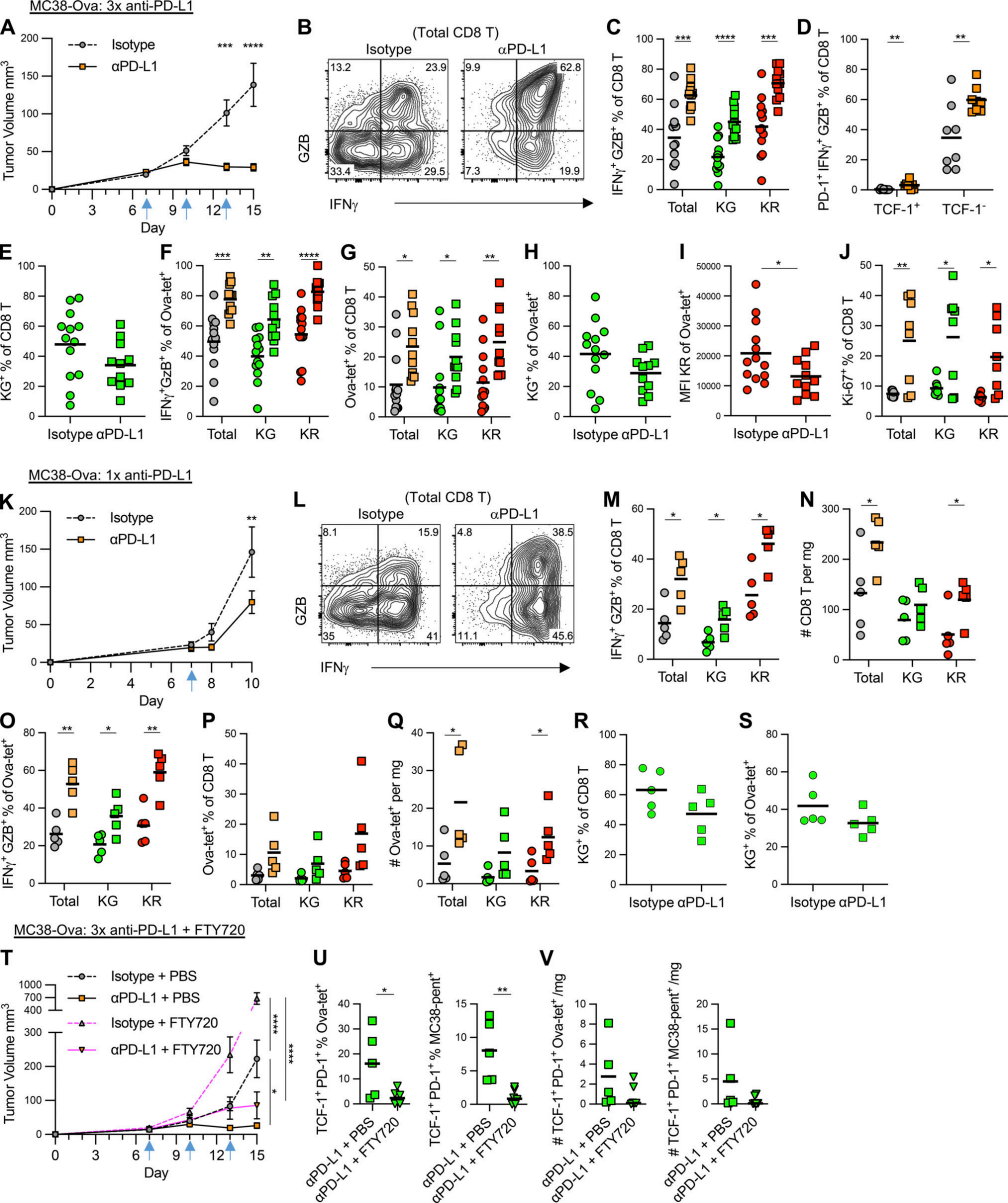
**Anti–PD-L1 Abs drive reinvigoration of exhausted effector cells alongside sustained activation of newly arrived populations.** MC38-Ova tumors treated with three doses (at day 7, 10 and 13; blue arrows) of anti–PD-L1 (*n* = 11) or isotype control Abs (*n* = 13) pooled from two independent experiments (A–J), or one dose (at day 7, blue arrow) of anti–PD-L1 (*n* = 5) or isotype control Abs (*n* = 5) from one independent experiment (K–S), or combinations of three doses (at day 7, 10, and 13; blue arrows) of anti–PD-L1 or isotype control Abs with five doses (at day 6, 8, 10, 12, and 14) of FTY720 or PBS (*n* = 5 for isotype control + PBS control; *n* = 5 for anti–PD-L1 + PBS control; *n* = 6 for isotype control + FTY720; *n* = 6 for anti–PD-L1 + FTY720 from one independent experiment; T–V). **(A)** Mean growth curve of tumors, error bars show SEM, arrows mark days of Ab dosing. **(B)** Representative flow cytometry plots showing expression of IFNγ and Granzyme B (GZB) by CD8 T cells in tumor. **(C)** Proportion of total, Kaede Green^+^, and Kaede Red^+^, CD8 T cells expressing both IFNγ and Granzyme B. **(D)** Proportion of PD-1^+^ TCF-1^+^ IFNγ^+^ GZB^+^ and PD-1^+^ TCF-1^−^ IFNγ^+^ GZB^+^ cells amongst CD8 T cells (*n* = 8 for each group). **(E)** Proportion of Kaede Green^+^ cells amongst CD8 T cells. **(F)** Proportion of total, Kaede Green^+^ and Kaede Red^+^ Ova-specific CD8 T cells (Ova-tet^+^) expressing both IFNγ and Granzyme B. **(G)** Proportion of Ova-specific cells (Ova-tet^+^) amongst the total, Kaede Green^+^, and Kaede Red^+^ CD8 T cell compartment. **(H)** Proportion of Kaede Green^+^ cells amongst Ova-specific CD8 T cells (Ova-tet^+^). **(I)** MFI of Kaede Red^+^ Ova-specific CD8 T cells. **(J)** Proportion of total, Kaede Green^+^ and Kaede Red^+^ CD8 T cells expressing Ki-67 (*n* = 8 for each group). **(K)** Mean growth curve of tumors, error bars show SEM, arrows mark days of Ab dosing. **(L)** Representative flow cytometry plots showing expression of IFNγ and Granzyme B (GZB) by CD8 T cells in tumor. **(M)** Proportion of total, Kaede Green^+^, and Kaede Red^+^ CD8 T cells expressing both IFNγ and Granzyme B. **(N)** Number of total, Kaede Green^+^, and Kaede Red^+^ CD8 T cells per mg tumor weight. **(O)** Proportion of total, Kaede Green^+^, and Kaede Red^+^ Ova-specific CD8 T cells (Ova-tet^+^) expressing both IFNγ and Granzyme B. **(P)** Proportion of Ova-specific cells (Ova-tet^+^) amongst the total, Kaede Green^+^, and Kaede Red^+^ CD8 T cell compartment. **(Q)** Number of total, Kaede Green^+^, and Kaede Red^+^ Ova-specific CD8 T cells per mg tumor weight. **(R)** The proportion of Kaede Green^+^ cells amongst CD8 T cells. **(S)** The proportion of Kaede Green^+^ cells amongst Ova-specific CD8 T cells. **(T)** Mean growth curve of tumors, error bars show SEM. **(U)** The proportion of TCF-1^+^ PD-1^+^ amongst the total, Kaede Green^+^, and Kaede Red^+^ Ova-specific (Ova-tet^+^) or MC38-neo-Ag–specific (MC38-pent^+^) CD8 T cell compartment. **(V)** The number per mg tumor of TCF-1^+^ PD-1^+^ Ova-specific or MC38-neo-Ag–specific CD8 T cells, TCF-1^+^ PD-1^+^ Kaede Green^+^ Ova-specific or MC38-neo-Ag–specific CD8 T cells and TCF-1^+^ PD-1^+^ Kaede Red^+^ Ova-specific or MC38-neo-Ag–specific CD8 T cells. All bars on graphs represent the mean. Statistical significance was tested with two-way ANOVA with Sidak’s multiple comparisons test (A, K, and T), unpaired multiple *t* tests (C, D, F, G, J, and M–Q) and unpaired two-tailed *t* test (E, H, I, R, S, U, and V): *, P ≤ 0.05; **, P ≤ 0.01; ***, P ≤ 0.001; ****, P ≤ 0.0001.

**Figure S5.**
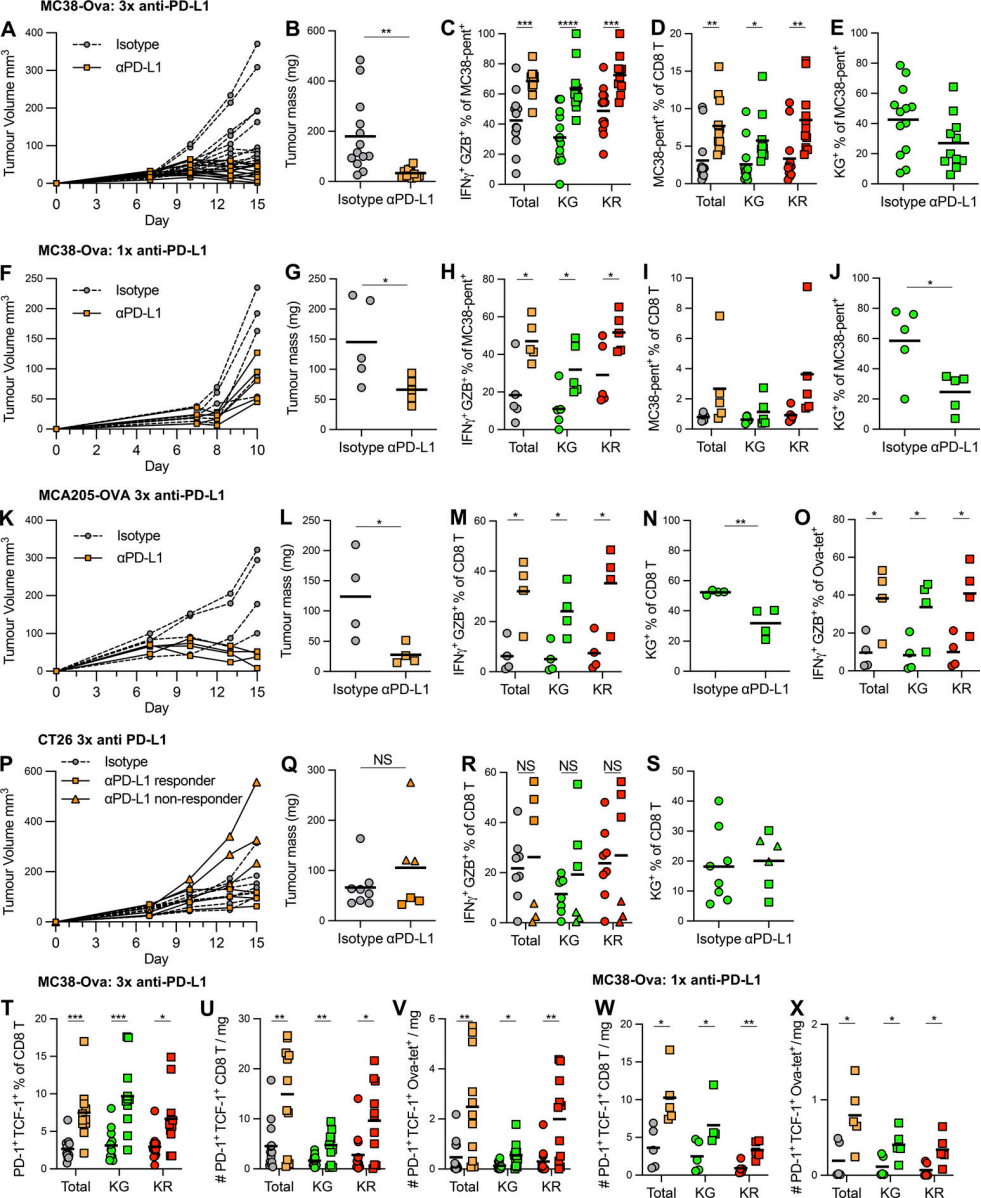
**Anti–PD-L1 Abs drive reinvigoration of exhausted effector cells alongside sustained activation of newly arrived populations in multiple tumor models.** MC38-Ova tumors treated with three doses of anti–PD-L1 (*n* = 11) or isotype control Abs (*n* = 13; A–E and T–V) pooled from two independent experiments, or one dose anti–PD-L1 (*n* = 5) or isotype control Abs (*n* = 5) from one independent experiment (F–J and W–X); MCA205-Ova tumors treated with three doses of anti–PD-L1 (*n* = 4) or isotype control Abs (*n* = 4) from one independent experiment (K–O); CT26 tumors treated with three doses of anti–PD-L1 (*n* = 6 including three responders and three non-responders) or isotype control Abs (*n* = 8) from one independent experiment (P–S). The TCF-1^+^ PD-1^+^ CD8 T cell compartment was assessed in T–X. **(A)** Individual tumor growth curves. **(B)** Tumor mass (mg) upon tissue harvest. **(C)** Proportion of total, Kaede Green^+^ and Kaede Red^+^ neo-Ag–specific CD8 T cells expressing both IFNγ and Granzyme B. **(D)** Proportion of neo-Ag–specific cells amongst the total, Kaede Green^+^, and Kaede Red^+^ CD8 T cell compartment. **(E)** Proportion of Kaede Green^+^ cells amongst neo-Ag–specific CD8 T cells. **(F)** Individual tumor growth curves. **(G)** Tumor mass (mg) upon tissue harvest. **(H)** Proportion of total, Kaede Green^+^, and Kaede Red^+^ neo-Ag–specific CD8 T cells expressing both IFNγ and Granzyme B. **(I)** Proportion of neo-Ag–specific cells amongst the total, Kaede Green^+^ and Kaede Red^+^ CD8 T cell compartment. **(J)** Proportion of Kaede Green^+^ cells among neo-Ag–specific CD8 T cells. **(K)** Individual tumor growth curves. **(L)** Tumor mass (mg) upon tissue harvest. **(M)** Proportion of total, Kaede Green^+^ and Kaede Red^+^ CD8 T cells expressing both IFNγ and Granzyme B. **(N)** Proportion of Kaede Green^+^ cells amongst CD8 T cells. **(O)** Proportion of Ova-specific T cells expressing both IFNγ and Granzyme B amongst the total, Kaede Green^+^, and Kaede Red^+^ CD8 T cell compartment. **(P)** Individual tumor growth curves. **(Q)** Tumor mass (mg) upon tissue harvest. **(R)** Proportion of total, Kaede Green^+^ and Kaede Red^+^ CD8 T cells expressing both IFNγ and Granzyme B. **(S)** Proportion of Kaede Green^+^ cells amongst CD8 T cells. **(T)** The proportion of TCF-1^+^ PD-1^+^ amongst the total, Kaede Green^+^, and Kaede Red^+^ CD8 T cell compartment. **(U)** The number per mg tumor of TCF-1^+^ PD-1^+^ CD8 T cells, TCF-1^+^ PD-1^+^ Kaede Green^+^ CD8 T cells, and TCF-1^+^ PD-1^+^ Kaede Red^+^ CD8 T cells. **(V)** The number per mg tumor of TCF-1^+^ PD-1^+^ Ova-specific T cells, TCF-1^+^ PD-1^+^ Kaede Green^+^ Ova-specific T cells, and TCF-1^+^ PD-1^+^ Kaede Red^+^ Ova-specific T cells. **(W)** The number per mg tumor of TCF-1^+^ PD-1^+^ CD8 T cells, TCF-1^+^ PD-1^+^ Kaede Green^+^ CD8 T cells and TCF-1^+^ PD-1^+^ Kaede Red^+^ CD8 T cells. **(X)** The number per mg tumor of TCF-1^+^ PD-1^+^ Ova-specific T cells, TCF-1^+^ PD-1^+^ Kaede Green^+^ Ova-specific T cells, and TCF-1^+^ PD-1^+^ Kaede Red^+^ Ova-specific T cells. All bars on graphs represent the mean. Statistical significance was tested with unpaired *t* test (B, G, J, L, N, Q, and S) and unpaired multiple *t* test (C, D, H, M, O, R, and U–X): *, P ≤ 0.05; **, P ≤ 0.01; ***, P ≤ 0.001; ****, P ≤ 0.0001.

By using direct photo-labeling, our data so far has assessed how CD8 T cells change after entry into the tumor and identified that, over several days, all CD8 T cells retained within this environment developed an exhausted phenotype. Targeting the PD-1:PD-L1 pathway not only drives the reinvigoration of exhausted T cells (Blackburn et al., 2008; Freeman et al., 2006) but also enhances T cell priming in the dLN to then drive superior T cell responses within the tumor (Dammeijer et al., 2020). Using our Kaede models to distinguish between newly recruited and retained cells within the tumor, we investigated whether these populations responded differently to anti–PD-L1 Abs. Kaede mice were grafted with MC38-Ova tumors and treated with three doses of anti–PD-L1 or isotype control Abs, then photoconverted, and analyzed 48 h later. This dosing regimen reproducibly impaired tumor growth (Figs. 8 A and S5 A), and the tumor mass upon tissue harvest was significantly reduced in treated animals (Fig. S5 B). The expression of Granzyme B and IFNγ was used as a measure of CD8 T cell function, and the proportion of CD8 T cells expressing both molecules was found to be significantly increased after anti–PD-L1 Ab administration (Fig. 8, B and C). Notably, both Kaede Green^+^ and Kaede Red^+^ CD8 T cells in the tumor showed a significant increase in the proportion expressing Granzyme B and IFNγ (Fig. 8 C). The vast majority of cells expressing Granzyme B and IFNγ lacked TCF-1 expression (Fig. 8 D). Furthermore, the proportion of Kaede Green^+^ CD8 T cells was not significantly different between isotype and PD-L1–treated mice, suggesting that anti–PD-L1 Abs did not result in enhanced recruitment of CD8 T cells into the tumor, at least between day 13–15 after tumor engraftment (Fig. 8 E). Analysis of Ag-specific CD8 T cell populations further confirmed that both the Kaede Green^+^ and Kaede Red^+^ subsets showed enhanced activation, and the proportion of these populations were significantly increased following administration of anti–PD-L1 Abs (Fig. 8, F and G; and Fig. S5, C and D). Again, the proportion of Kaede Green^+^ cells within Ag-specific population was not significantly different between the isotype and anti–PD-L1 treatment groups, arguing against the increased numbers of cells trafficking into the tumor (Figs. 8 H and S5 E). Rather, a significant reduction in the level of Kaede Red fluorescence suggested that the increased proportion of these cells reflected local proliferation (Fig. 8 I). Consistent with this, we observed an increased proportion of both Kaede Green^+^ and Kaede Red^+^ CD8 T cells expressing Ki-67 in the tumors of mice given anti–PD-L1 Abs (Fig. 8 J). Collectively, this analysis indicates that anti–PD-L1 Abs drive the enhanced activation of both newly recruited and exhausted CD8 T cells, which are increased through local expansion rather than through more cells recruited into the tumor.

Since tumor size was significantly different after three doses of anti–PD-L1 Abs and initial differences in recruitment may have ceased to be detectable at this stage of the response, we photoconverted tumors after only one dose of anti–PD-L1 Abs and analyzed 48 h later. A significant reduction in tumor size and mass was observed (Fig. 8 K; and Fig. S5, F and G), alongside increased activation of both newly-recruited and retained CD8 T cells within the tumor, including Ag-specific populations (Fig. 8, L and M; and Fig. S5 H). While the proportion and number of Ag-specific CD8 T cell populations increased (Fig. 8, N–Q; and Fig. S5 I), no difference in the proportion of Kaede Green^+^ cells was observed, again indicating that targeting PD-L1 had not substantially enhanced the numbers of CD8 T cells recruited into the tumor (Fig. 8, R and S; and Fig. S5 J).

To extend these observations to further tumor models, Kaede mice bearing either MCA205 or CT26 tumors were treated with three doses of anti–PD-L1 Abs. In all mice with MCA205 tumors, we again observed significantly impaired tumor growth and enhanced effector functions amongst both Kaede Green^+^ and Kaede Red^+^ CD8 T cells (Fig. S5, K–O). While only some mice showed impaired growth of CT26 tumors upon anti–PD-L1 Abs (Fig. S5, P and Q), in these responders, enhanced activation of both Kaede Green^+^ and Kaede Red^+^ CD8 T cells was evident (Fig. S5 R). Notably, although, the proportion of Kaede Green^+^ CD8 T cells within responder or non-responder tumors was comparable with isotype controls, again indicating that the proportion of CD8 T cells recruited into the tumor was not altered by the targeting PD-L1 tumor (Fig. S5 S), we further observed that anti–PD-L1 Abs increased both the proportion and number of TCF-1^+^ PD-1^+^ CD8 T cells within the tumor (Fig. S5, T–X). Finally, to assess the functional contribution made by newly recruited CD8 T cells, mice bearing MC38-Ova tumors were treated with anti–PD-L1 or isotype control Abs in combination with the S1PR agonist FTY720 (Brinkmann et al., 2004; Matloubian et al., 2004). Treatment with FTY720 reduced the ability of anti–PD-L1 Abs to restrain tumor growth, providing functional evidence of the contribution made by CD8 T cells newly recruited into the tumor (Fig. 8 T). Given that the proportion of Kaede Green^+^ CD8 T cells in the tumor was not significantly increased with anti–PD-L1 Abs, our data are consistent with the enhanced responsiveness of the newly recruited cells rather than overt increases in the number of CD8 T cells trafficking into the tumor. A significant reduction in the proportion of Ag-specific Kaede Green^+^ TCF-1^+^ PD-1^+^ CD8 T cells in the tumor was observed as a result of FTY720 treatment; however, these were very small populations of cells, and the total numbers were not significantly reduced (Fig. 8, U and V).

Collectively, our data reveal that targeting PD-L1 enhances the anti-tumor T cell response through rejuvenating activated/exhausted CD8 T cells retained within the tumor, as well as through the actions of newly recruited CD8 T cells. We observed no clear evidence that more CD8 T cells were recruited into the tumor, rather, our data indicated that anti–PD-L1 Abs resulted in the enhanced functions of cells arriving into the tumor, and these cells, combined with actions of reinvigorated CD8 T cells retained within the tumor, limited tumor growth.

## Discussion

Here, we provide the first in vivo analysis of T cell recruitment and retention within tumors using photoactivation to directly label the cells in situ and then track changes over time. This approach enabled the definitive identification of cells newly entering the tumor, which in turn facilitated the investigation of how cells changed when retained within this environment. Through tracking T cell egress from the tumor, we assessed the fate of different intratumoral T cell subsets, discovering that TCF-1^+^ T cell populations, including the PD-1^+^ “stem-like” population, escape the tumor and return to lymphoid tissue through the lymphatic drainage. Furthermore, our data clearly shows that CD8 T cells rapidly become exhausted within the tumor and that in the preclinical models used in our studies, exhausted cells comprise the vast majority of the CD8 T cell population retained within the tissue. Utilizing the novel models described here, we further determined that the blockade of PD-L1 reinvigorates exhausted CD8 T cells in the tumor, while also enhancing the functions of effector cells that recently entered the tissue.

Essential to the success of our experimental approach was the simultaneous labeling of all host cells within the tumor. Previous efforts to photoconvert very small tumors within the ear pinnae of Kaede mice (Torcellan et al., 2017) failed to label all cells, thus limiting the study to T cell egress. Complete labeling of all host cells within the tumor enabled us to define the phenotype of the cells as they enter the tumor and thus changes over time could be determined. While our analysis was restricted to a time frame of only a few days, the extent to which the TIL compartment changed was striking with all TIL populations increasing ∼10-fold in number through constant recruitment into the tissue. Since we were only able to fully photoconvert tumors up to approximately 8 mm in diameter, we were frustrated in our efforts to investigate whether the rate of lymphocyte influx was dependent upon the stage of tumor development. Refined labeling approaches may enable larger tumors to be studied, which might better represent the more established tumors identified in patients. Further integration of spatial analysis approaches to assess infiltrating and retained TIL populations alongside better characterization of different microenvironments within the tumor may also provide new insights into the establishment of immunologically “hot” and “cold” tumors, facilitating new therapeutic avenues.

Although the exhausted CD8 T cell population of tumors has been thoroughly characterized at the transcriptomic level (Jansen et al., 2019; Kurtulus et al., 2019; Siddiqui et al., 2019; Singer et al., 2016), the rate at which an exhausted phenotype occurs in vivo has been challenging to determine. Single-cell transcriptomic analysis of the newly entering CD8 T cells indicated a mix of naive, memory, and stem-like cells and notably, the absence of clear effector populations, strikingly similar to the characterization of PD-1^−^ CD8 T cell populations within the tumor (Kurtulus et al., 2019). Trajectory analysis integrated with the temporal sampling indicated the rapid emergence of the effector program once the cells were recruited into the tumor. However, our data reveal that within a time frame of a few days, these effector cells become exhausted. The most striking observation from our transcriptomic data was the homogenous nature of CD8 T cells retained within the tumor for over 72 h. The CD8T5 cluster bore multiple hallmarks of exhaustion and accounted for the vast majority of the retained CD8 T cell population, while the clusters dominated by newly entering cells (CD8T1 and CD8T2) were absent in the Kaede Red^+^ 72 h sample. While our data obviously does not exclude the differentiation of TCF-1^+^ CD8 T cell populations in the tumor, it does demonstrate that these phenotypes are not maintained over time. Indeed, these populations comprised the majority of the CD8 T cells egressing the tumor, indicating that escape from the tumor enables the preservation of some T cell subsets, particularly those that can potentially support protective immunity.

A key unanswered question arising from our study is whether the stromal compartment of the tumor might alter the T cell recruitment dynamics observed. Models such as MC38 and CT26 have a much lower cancer-associated fibroblast (CAF) composition than human tumors, and seeding of these tumor cell line models with fibroblasts to enhance CAF numbers significantly reduced CD8 T cell numbers in the tumor (Ford et al., 2020). This suggests that the ability of T cell populations to be retained in the tumor may be dictated by the extent and the nature of the CAFs present, potentially creating or limiting the recruitment and survival factors forming certain niches. Of particular note in this regard is the TCF-1^+^ PD-1^+^ population, which is thought to be tissue-resident within LNs in the context of LCMV infection (Im et al., 2020), but able to migrate into the tumors (Connolly et al., 2021). Our data indicate that the pool of TCF-1^+^ PD-1^+^ cells within the tumor is constantly replenished by newly entering cells. While other recent studies also concluded this (Connolly et al., 2021), our data provide direct evidence that these cells egress the tumor and return to the dLN. This escape of TCF-1^+^ PD-1^+^ cells from the tumor environment enables the preservation of this population despite chronic Ag exposure within the tumor driving differentiation to effector states. These observations are analogous to recent descriptions of the recirculation of TCF-1^+^ PD-1^+^ CD8 T cells through lymphoid tissue in type 1 diabetics (Abdelsamed et al., 2020).

Targeting of immune checkpoints is thought to reinvigorate exhausted CD8 T cells (Wherry et al., 2007). Our data distinguishing newly entering versus retained T cell populations further indicates that blockade of PD-L1 ensures sustained the activation of the effector CD8 T cells recently entering the tumor. Given the restricting PD-L1 availability only within the dLN restricted tumor growth (Dammeijer et al., 2020), the enhanced responsiveness of the Kaede Green^+^ cells may reflect a superior activation in this environment that better equips them to sustain their functions upon tumor entry. However, we cannot exclude that the newly entering CD8 T cells encounter a more permissive environment within the tumor as a result of anti–PD-L1 Ab administration. TCR-based tracking of T cell clones after anti–PD-1 therapy indicated that the enhanced T cell response to checkpoint blockade derives from T cell clones recently entering the tumor (Yost et al., 2019). Determining whether specific mechanisms can enhance the recruitment or retention of TCF-1^+^ PD-1^+^ CD8 T cells may provide new ways to reinforce effector responses and synergize with targeting different inhibitory receptors.

In common with the behavior of different CD8 T cell subsets, our data also indicated that the tumor Treg compartment is a mix of cells trafficking through the tissue alongside the specific retention and differentiation of some populations within the tumor. The majority of Tregs, both entering and exiting the tumor expressed NRP-1, potentially suggest a thymic origin, although this requires further validation. A small NRP-1^−^ Treg population was also amongst the newly entering Treg pool, potentially representing peripherally induced Tregs recognizing tumor neo-Ags. Further experiments that track Ag-specific Tregs are required to better assess the Treg populations beyond surface phenotypes that fail to conclusively define their origin. Our analysis does clearly indicate that the expression of LAG-3 is a robust marker for Tregs retained within the tumor, and the data presented here provide a new resource to help define the Treg compartment of tumors. Building on these data to identify mechanisms controlling Treg retention within the tumor is an obvious further advance with therapeutic potential.

In summary, here we have provided the first detailed in vivo analysis of T cell recruitment and retention within preclinical tumor models. Our data reveal a highly dynamic system containing migratory TIL subsets alongside bona fide tumor resident populations. The intratumoral effector response is sustained by continuous recruitment of TCF-1^+^ PD-1^+^ CD8 T cells from peripheral sources, which in turn circulate back to draining lymphoid tissue to escape chronic Ag exposure.

## Materials and methods

### Mice

C57BL/6 Kaede (Tomura et al., 2008) and Kaede × OTI mice were maintained and bred at the University of Birmingham Biomedical Services Unit. BALB/c Kaede mice were acquired from the RIKEN BioResource Center, Experimental Animal Division. Mice were culled between the ages of 7 and 15 wk. Animals were used in accordance with Home Office guidelines at the University of Birmingham under a Project Licence awarded to D.R. Withers and approved by the University of Birmingham Animal Welfare and Ethical Review Body. Mice were housed at 21 ± 2°C, 55% humidity (±10%), with 12 h light dark/cycle in 7–7 individually ventilated caging with environmental enrichment of plastic houses plus paper bedding.

### Subcutaneous tumor models

MC38 murine colon adenocarcinoma cells (kindly provided by Dr. Gregory Sonnenberg; Weill Cornell Medicine, New York, NY), MC38-Ova murine colon adenocarcinoma cells (obtained from AstraZeneca), MCA205-Ova murine fibrosarcoma cells (obtained from AstraZeneca), and CT26 murine colorectal carcinoma cells (kindly provided by Professor Tim Elliott, University of Oxford, Oxford, UK) were cultured in RPMI supplemented with 2 mM L-glutamine (21875034; Thermo Fisher Scientific), 10% FBS (F9665; Sigma-Aldrich), and penicillin–streptomycin (P4333; Sigma-Aldrich) at 37°C and with 5% CO_2_. Cells grown in the log phase were then harvested and suspended in Dulbecco’s PBS (D8662; Sigma-Aldrich), and 100 µl of cell suspension containing 2.5 × 10^5^ cells (MC38; MC38-Ova or CT26) or 5 × 10^5^ cells (MCA205-Ova) were subcutaneously injected into mice in the pre-shaved left flank area under anesthesia via 2% gaseous isoflurane. Where one dose of anti–PD-L1 Abs was used, 1 × 10^6^ MC38-Ova cells were injected subcutaneously. Tumor size was periodically measured with a digital Vernier caliper, and the volume was expressed in cubic millimeters using the formula *V* = 0.5 × *a* × *b*^2^, where *a* and *b* are the long and short diameters of the tumor, respectively. Tumor weight was measured at the endpoint of experiment.

### Labeling of tumor compartment by “photoconversion”

After tumor inoculation, palpable tumor usually develops within a week. On day 11, the tumor was exposed to a 405-nm wavelength LED light from a fixed distance of 1 cm using a Dymax BlueWave QX4 system (DYM41572; Intertronics) outfitted with an 8 mm focusing lens for a total of 3 min at 50% of full power with a 5-s break for every 20 s. Black cardboard was used to shield the rest of the mouse. Confirmation that this approach resulted in all cells within the tumor expressing the converted “Kaede Red” version of the fluorochrome was achieved through analysis immediately (0 h) after labeling. While all converted cells exhibited Kaede Red fluorescence, most cells also were weakly Kaede Green^+^, likely reflecting the expression of the new Kaede protein subsequent to photoconversion. Regardless, the distinct Kaede Red^+^ expression profile enabled discrimination of the newly entering and resident populations by both flow cytometry and immunofluorescence.

### Intravenous injections of anti-CD45 Abs

To distinguish tissue-infiltrating leukocytes from circulating leukocytes present in vasculature, mice were injected intravenously under anesthesia with 2 μg BUV395-conjugated CD45 Ab (clone 30-F11; BD) diluted in 200 μl PBS via tail vein and then were culled after 2 min.

### Administration of anti–PD-L1 Abs

For three-dose experiments, anti–PD-L1 mouse IgG1 (Clone 80, SP16-260; AstraZeneca) or NIP228 isotype control mouse IgG1 (SP16-017; AstraZeneca) were administered by intraperitoneal injection using three doses of 200 µg diluted in 200 µl PBS (10 mg/kg body weight) on day 7, 10, and 13 following tumor injection in C57BL/6 or BALB/c Kaede mice. Some mice also received additional intraperitoneal injections with FTY720 (SML0700; Sigma-Aldrich) using five doses of 40 µg diluted in 200 µl PBS (2 mg/kg body weight) or PBS control on day 6, 8, 10, 12, and 14 following tumor injection. Tumor photoconversion was performed on day 13 and tumors were measured by a caliper on day 7, 10, 13, and 15. Mice were sacrificed for analysis on day 15. For one-dose experiments, anti–PD-L1 or NIP228 isotype control mouse IgG1 were administered by intraperitoneal injection using one dose of 200 µg diluted in 200 µl PBS (10 mg/kg body) on day 7 following MC38-Ova injection in C57BL/6 Kaede mice. Tumor photoconversion was performed on day 8 and tumors were measured by a caliper on day 7, 8, and 10. Mice were sacrificed for analysis on day 10.

### Cell isolation and flow cytometry

The tumor was cut into small pieces and incubated with 1 mg/ml Collagenase D (11088882001; Roche) and 0.1 mg/ml DNase I (101104159001; Roche) in a volume of 1.2 ml RPMI media at 37 °C on a thermomixer (Eppendorf) for 20 min. After incubation, undigested debris was removed by filtering the sample through a 70-µm strainer. Tumor-draining LN (i.e., left inguinal LN) was cleaned and teased in RPMI 1640 medium (Thermo Fisher Scientific) and crushed through a 70-μm filter. Spleen was smashed through a 70-µm strainer followed by incubation with 5 ml red blood cell lysis buffer Gey’s solution on ice for 5 min to lyse the red blood cells. Cells were harvested by centrifuging the samples at 400 *g* at 4°C for 5 min and resuspended in FACS staining buffer (2% FBS, 2 mM EDTA in PBS) and subjected to Fc block with anti-CD16/32 (1:200, clone 2.4G2; BioLegend) diluted in FACS staining buffer on ice for 10 min before staining for Live/Dead Fixable Dead cell Stain Kits (1:1,000, L34960; Thermo Fisher Scientific) and surface markers diluted in FACS staining buffer on ice for 30 min. Cells were then fixed with BD Cytofix fixation buffer (554655; BD) for 40 min and stained for intracellular markers diluted in eBioscience permeabilization buffer (00-8333-56; Thermo Fisher Scientific) at room temperature overnight. To identify Ag-specific CD8 T cells, the cell suspension was incubated with APC-labeled MC38 neo-Ag pentamer H-2K^b^-KSPWFTTL (F828-4A-G; ProImmune) diluted to 1:10 in staining buffer or BV421-labeled Ova-Ag tetramer H-2K^b^-SIINFEKL (National Institutes of Health) diluted to 1:200 in staining buffer for 1 h at 37°C prior to surface marker staining. To assess cytokine production, cell suspension was stimulated with 50 ng/ml Phorbol 12-myristate 13-acetate (PMA, P1585; Sigma-Aldrich) and 1.5 µM ionomycin (I0634; Sigma-Aldrich) for 3 h in the presence of 10 µg/ml brefeldin A (B6542; Sigma-Aldrich) prior to staining. To assess the absolute cell numbers, 1 × 10^4^ counting beads (ACBP-100-10; Spherotech) were added to each stained sample at the last step. Data were acquired on the BD LSR Fortessa X-20 (BD) using FACSDiva 8.0.2 software (BD) and analyzed with FlowJo v10 (BD).

The Abs raised against the following mouse Ags were used: B220 PE/Dazzle 594 (1:200; RA3-6B2; BioLegend), CD3 AF700 or BUV737 or BUV395 or BV605 (1:200; clone 145-2C11 or 17A2; eBioscience, BD Biosciences, or BioLegend), CD4 BUV395 (1:100; clone RM4-5; BioLegend), CD8a BV510 or BV711 (1:200; clone 53-6.7; BioLegend), CD11b PE/Cy7 or PE/Dazzle 594 (1:400; clone M1/70; BioLegend), CD11c BV786 or PE/Cy7 or PE/Dazzle 594 (1:200; clone N418; BioLegend), CD25 BV650 or BV605 (1:200; clone PC61; BioLegend), CD29 Super Bright 702 (1:200; clone HMb1-1; eBioscience), CD39 Super Bright 600 (1:200; clone 24DMS1; eBioscience), CD45 APC (1:200; clone 30-F11; BioLegend), CD45.2 BUV395 (1:200; clone 104; BD), CD45.2 BV785 (1:200; clone 104; BioLegend), CD62L BV711 or BV650 (1:200; clone MEL/14; BioLegend), CD69 BV711 or BV605 (1:200; clone H1.2F3; BioLegend), Foxp3 e450 or APC (1:200, clone FJK-16s; eBioscience), Granzyme B AF700 (1:200; clone QA16A02; BioLegend), ICOS BV510 (1:200; clone C398.4A; Biolegend), IFNγ BUV737 (1:200; clone XMG1.2; BD), IL-10 PE-Cy7 (1:200; clone JES5-16E3; BioLegend), Ki-67 PE-Cy7 (1:200; clone SolA15; eBioscience), LAG-3 BV785 (1:200; clone C9B7W; Biolegend), LAP-1 (1:200; clone TW7-16B4; eBioscience), MHC-II (1-A/I-E) BV510 (1:200, clone M5.114.15.2; BioLegend), Neuropilin-1 (NRP-1) purified and raised in goat (1:200; AF556; R&D System), NK1.1 BV650 (1:200; clone PK136; BD Biosciences), PD-1 BV421 or APC or BV605 (1:200; clone 29F.1A12; BioLegend), and TCF-1 PE-Cy7 (1:200, clone C63D9; Cell Signalling). AF647-conjugated donkey anti-goat IgG secondary Ab (1:500; A-21447; Thermo Fisher Scientific) was used to amplify and label Neuropilin-1 staining.

### Immunofluorescence staining

Tumor tissue with the overlying skin was collected, fixed with BD Cytofix fixation buffer (554655; BD) overnight, then preserved with 30% sucrose (S8501; Sigma-Aldrich) overnight prior to embedding and freezing in O.C.T. (Tissue-Tek). Then 8-μm-thick frozen sections were cut in a cryostat (OTF5000; Bright), fixed with BD Cytofix fixation buffer, and then stained with a far red DNA dye Draq5 (1:1,000, 424101; BioLegend). Images were taken using a Zeiss LSM 880 microscope (Zeiss) and analyzed with ImageJ.

### Bulk RNA-seq

MC38 tumors were inoculated in age- and gender-matched C57BL/6 Kaede mice. Some of these tumors were photoconverted on day 10 as described above and collected 5 h after photoconversion. Tumors that were not photoconverted were also collected at the same time for comparison. Samples were preserved in RNAlater stabilization solution (AM7020; Thermo Fisher Scientific) and then homogenized. RNA was extracted using a Qiacube with the RNAmini kit (Qiagen). Libraries were produced using Illumina Trueseq stranded total library prep kit. Sequencing was carried out on a Novaseq6000 using a 2 × 150 read.

### Single-cell isolation, library construction, and sequencing

MC38 tumors were inoculated in age- and gender-matched C57BL/6 Kaede mice and photoconverted on day 11 as described above. Mice with tumors in similar sizes were collected 24 or 72 h after tumor photoconversion. After tumor digestion as detailed above, cell suspensions were stained for CD45 BV650 (1:100, clone 30-F11; BioLegend), CD11b PE-Cy7 (1:400; clone M1/70; BioLegend), Ter119 PerCP-Cy5.5 (1:200; clone Ter119; BioLegend), Live/dead APC-Cy7 1:2,000 on ice for 30 min. Tumor-infiltrating lymphocytes (live CD45^+^Ter119^−^CD11b^−^) were sorted with a FACS Aria II Cell Sorter (BD) into two groups based on the presence or absence of Kaede red at each time point, namely G24 (Kaede Green 24 h with 9,500 cells), R24 (Kaede Red 24 h with 30,000 cells), G72 (Kaede Green 72 h with 15,000 cells), and R72 (Kaede Red 72 h with 22,000 cells).

Gene expression libraries were prepared from single cells using the Chromium Controller and Single Cell 3ʹ Reagent Kits v2 (10× Genomics, Inc.) according to the manufacturer’s protocol (CG00052; Rev B). The resulting sequencing libraries comprised standard Illumina paired-end constructs flanked with P5 and P7 sequences. The 16 bp 10× Barcode and 10 bp UMI were encoded in Read 1, while Read 2 was used to sequence the cDNA fragment. Sample index sequences were incorporated as the i7 index read. Paired-end sequencing (26:98) was performed on the Illumina NextSeq500 platform using NextSeq 500/550 High Output v2.5 (150 Cycles) reagents. The .bcl sequence data were processed for QC purposes using bcl2fastq software (v. 2.20.0.422), and the resulting.fastq files were assessed using FastQC (v. 0.11.3), FastqScreen (v. 0.9.2), and FastqStrand (v. 0.0.5) prior to pre-processing with the CellRanger pipeline (Zheng et al., 2017).

### Processing of scRNA-seq data

Single-cell gene expression data from cellranger output was analyzed using standard Seurat-inspired scanpy (v.1.4.5.post2) workflow (Stuart et al., 2019; Wolf et al., 2018). Doublet detection was performed using scrublet (v0.2.1; Wolock et al., 2019) with adaptations outlined in Popescu et al. (2019). Briefly, after scrublet was performed, the data was iteratively sub-clustered and a median scrublet score for each sub-cluster was computed. Median absolute deviation scores were computed from the cluster scrublet scores and a one-tailed *t* test was performed with Benjamini–Hochberg correction (Benjamini and Hochberg, 1995) applied, and cells with significantly outlying cluster scrublet scores (Benjamini–Hochberg P value <0.1) were flagged as potential doublets. The data was then processed using scanpy with standard quality control steps; cells were filtered if the number of genes >2,500 or <200. Percentage mitochondrial content cut-off was set at <5%. Genes were retained if they are expressed by at least three cells. Gene counts for each cell were normalized to contain a total count equal to the median of total counts in cells before normalization. This led to a working dataset of 3,654 cells. Highly variable genes were selected based on the following parameters: minimum and maximum mean expression are ≥0.0125 and ≤3 respectively; minimum dispersion of genes = 0.5. The number of PCs used for neighborhood graph construction and dimensional reduction was set at 50. Clustering was performed using the Leiden algorithm (Traag et al., 2019) with the resolution set at 1.0. Uniform Manifold Approximation and Projection (UMAP; v3.10.0; McInnes et al., 2018) was used for dimensional reduction and visualization, the minimum distance was set at 0.3, and all other parameters as per default settings in scanpy.

### Differential gene testing

Differential gene testing was performed using the Wilcoxon test rank-sum test implemented in scanpy’s rank_genes_groups module.

### Gene set testing

Gene set testing was performed using scanpy’s tl.score_genes tool. Gene sets from the respective studies were downloaded from Gene Expression Omnibus accession numbers GSE84015 (Im et al., 2016), GSE140430 (Jansen et al., 2019), and GSE86042 (Singer et al., 2016).

### Trajectory analyses and TF enrichment analysis

Cell trajectory analyses were performed using partition-based graph abstraction (PAGA; Wolf et al., 2019) and palantir (Setty et al., 2019). TF and regulon enrichment were performed using pyscenic (Van de Sande et al., 2020) with genes found to be significantly differentially expressed along pseudotime using tradeSeq (Van den Berge et al., 2020). Regulon specificity/activity scores were calculated with pyscenic’s regulon_specificity_scores.

### Statistical analysis

Mice were gender-matched (female) and tumor growth curves for anti–PD-L1 and isotype control groups were analyzed using two-way ANOVA followed by Sidak’s multiple comparisons test and are presented as mean values ± SEM. Unpaired multiple *t* tests were used when multiple parameters were compared between anti–PD-L1 and isotype groups. Multiple comparisons in untreated animals were performed using an ordinary one-way ANOVA, followed by Tukey’s multiple comparisons test. Unpaired two-tailed Student’s *t* test was used if two groups with normal distributions were compared, while pairs of samples without normal distribution were compared using an unpaired two-tailed Mann-Whitney test. Paired two-tailed *t* test was used if the parameters of different populations from the same animal were compared. P < 0.05 was considered statistically significant and denoted as follows: *, P < 0.05; **, P < 0.01; ***, P < 0.001; and ****, P < 0.0001. Where a P value is not indicated, no statistical difference was observed. Statistical analysis was performed using Graphpad Prism.

### Online supplemental material

Fig. S1 shows the gating strategy, enumeration of TILs within MC38, CT26, and MCA205 tumors, and the analysis of proliferation and vascular contamination. Fig. S2 shows bulk RNA-seq data from tumors 5 h after photoconversion (versus controls) to assess transcriptional changes induced by the photoconversion process. Fig. S3 shows an analysis of tumor Treg populations, complementing data in Fig. 3. Fig. S4 shows a detailed analysis of gene expression within the CD8 T cell clusters including DEGs and changes over pseudotime. Fig. S5 shows an analysis of the effects of anti–PD-L1 Abs in MC38, CT26, and MCA205 tumors.

## Data Availability

The scRNA-seq data are available in the ArrayExpress database (accession numbers E-MTAB-10176). The bulk RNA-seq data assessing the effect of photoconversion are available in the Gene Expression Omnibus database (accession number GSE193654).
